# ARHGAP44 gene: a cytoskeleton mobility-related modulator with implications in pan-cancer prognostic risk and immune regulation

**DOI:** 10.3389/fonc.2026.1721943

**Published:** 2026-03-31

**Authors:** Wenxia Ma, Jiayao Li, Huijun Yang, Xuzhi Wang, Siying Liu, Lei Miao, Ningning Shen, Zhiqing Yang, Lifang Gao, Chen Wang

**Affiliations:** 1 Department of Pathology, Second Hospital of Shanxi Medical University, Taiyuan, Shanxi, China; 2 Department of Pathology, Second Clinical Medical College of Shanxi Medical University, Taiyuan, Shanxi, China; 3 Department of Pathology, Public Health School of Shanxi Medical University, Taiyuan, Shanxi, China; 4 Department of Pathology, Basical College of Shanxi Medical University, Taiyuan, Shanxi, China

**Keywords:** ARHGAP44, cytoskeleton, drug target, immune regulation, prognosis risk

## Abstract

**Introduction:**

Rho GTPases have been a well-known family of small G proteins that regulate cellular cytoskeleton dynamics and are involved in multiple critical steps of cancer progression. However, ARHGAP44 gene, a member of GAP proteins that regulate the Rho GTPases cycling between their active GTP-bound and inactive GDP-bound states, remains poorly understood in terms of its role in cancer development. This study aims to analyze the functions of ARHGAP44 gene in a broad spectrum of human cancers, thus aiding in the better understanding of the collaborative network of cytoskeleton-related genes in cancers.

**Methods:**

The study started with the analysis of the genetic characteristics of ARHGAP44 gene, followed by its expression patterns, frequent alterations as well as survival prediction value in a broad spectrum of human cancers. Furthermore, the probable reasons for the aberrant changed expression of ARHGAP44 in cancers compared to corresponding normal control samples were investigated. Moreover, the correlation of ARHGAP44 with multiple critical clinical cancer parameters was performed in succession.

**Results:**

The basic genetic physicochemical properties of ARHGAP44 including its amino-acid composition, estimated molecular weight, and protein half-life were investigated. Then, genetic alteration analysis revealed that ARHGAP44 expression varies in human cancers, which was partly due to the modulation by DNA methylation and phosphorylation. Furthermore, ARHGAP44 gene was associated with multiple critical cancer traits including cancer stemness, cytoskeleton dynamics as well as immune infiltration in different human cancer types. Moreover, ARHGAP44 gene was also associated with the sensitivity of several chemotherapy-related drugs.

**Conclusions:**

Based on multiple analyses, some valuable strategies to guide the therapeutic orientation concerning the role of ARHGAP44 gene in human cancers were revealed, although more detailed experiments and clinical trials are obligatory to support further clinical medical applications of the gene, especially in each independent cancer type.

## 1 Introduction

Rho GTPases constitute a family of small G proteins well recognized for their capacity to regulate cellular cytoskeletal dynamics, not just in morphology maintenance, cell growth, and cell cycle progression, but also in cell migration, vesicle trafficking, and stem cell differentiation (1). Given that cancer mobility particularly cell migration and metastasis, represents a major cause of cancer-related mortality and involves intensive coordination of cytoskeleton movement (2), investigating the detailed biological functions of Rho GTPases in human cancers holds significant clinical relevance.

To date, 20 well characterized Rho GTPases have been classified into subfamilies as RHO (including RHOA, RHOB, and RHOC), RAC (containing RAC1, RAC2, RAC3, and RACG), CDC42 family (comprising of CDC42, RHOQ, and RHOJ), and RhoD/RhoF cluster (namely, RHOD and RHOF) (1). Among these 20 members, RHOA, RAC1, and CDC42 are the most thoroughly characterized. They have been reported to regulate cell cytoskeleton dynamics by activating multiple downstream effectors, such as protein kinases ROCK1 and ROCK2, which are both involved in modulating proteins that contribute to cellular actin filament stabilization and generation of actin-myosin contractile force (3, 4), as well as IQGAP family including IQGAP1, IQGAP2, and IQGAP3, which modulates cell shape and motility through regulating the equilibrium between G-actin and F actin (5–8). Additionally, WASP, WAVE proteins, and formins are effective RHO GTPase effectors that regulate critical cellular cytoskeleton dynamics including actin polymerization, cell motility, and mitosis (9–11).

A shared functional feature of these classic Rho GTPases proteins including RHOA,RAC1, and CDC42 is that they all cycling between an active GTP-bound and inactive GDP-bound state in cells (12). This cycling is tightly regulated by three classes of proteins including guanine nucleotide exchange factors (GEFs), GTPase activating proteins (GAPs), and guanine nucleotide dissociation inhibitors (GDIs). GEFs destabilize the interaction between Rho GTPases proteins and their bounding GDP, resulting in interacting with GTP. Meanwhile, GAPs promote the inactivation of Rho GTPases by stimulating their intrinsic GTP hydrolysis, driving the conversion from active GTP- bound to inactive GDP- bound states (13, 14). Moreover, GDIs function to maintain Rho GTPases in an inactive GDP-bound state and sequester them in the cytoplasm. The activity of a specific Rho GTPase is thus the product of a complex interplay among these regulatory factors (15, 16).

Numerous regulatory proteins including RHO GEFs, GAPs, and GDIs are known to be dysregulated via altered gene expression, genetic modifications, or different activity levels (17, 18). Such dysregulation can directly perturb Rho GTPase signaling, leading to diverse cellular outcomes (19). Over the years, hundreds of RHO GEFs and GAPs have been identified, with their expressions varying across different cancer types (15). However, detailed investigations of these regulatory RHO GEFs or GAPs remain notably scarce compared to the extensive analysis of the core Rho GTPases RHOA, RAC1, and CDC42 (20–22). Careful analysis of the modulating genes was also of potential clinical value and might shed promising insights for further understanding of the collaborative network of cellular mobility and cytoskeleton-related genes in cancers.

In the present study, we focused on ARHGAP44 (Rho GTPase-activating protein 44), a GAP protein that has been shown to act on at least CDC42 and RAC1 (23, 24). In neurons, ARHGAP44 has been implicated in spine formation and synaptic plasticity (25). Moreover, accumulating evidence links ARHGAP44 to the survival of several cancers, including osteosarcoma (26, 27), hepatocellular carcinoma (28) and melanoma (29). Nevertheless, the comprehensive role of ARHGAP44 in human cancers, especially its detailed function and underlying mechanisms, remains poorly understood.

Using the TCGA pan-cancer data, we conducted a comprehensive analysis of ARHGAP44 in human cancers, especially its expression patterns and clinical traits across different cancers. To be more specific, the study started with the investigation of basic genetic information and physicochemical properties of ARHGAP44, and then the expression patterns, genetic alterations as well as prognostic value in pan-cancer, followed by potential post-transcription modification including phosphorylation and methylation. Furthermore, clinical traits including association with cancer stemness, cytoskeleton dynamics, immune infiltration, and drug sensitivity were preliminarily analyzed. The results shall provide promising insights for understanding the working mechanism of ARHGAP44 in human cancers, thereby facilitating the identification of potential novel prognostic biomarkers and therapeutic targets.

## 2 Methods

### 2.1 Data source: TCGA pan-cancer gene profiles

TCGA pan-cancer data were retrieved from UCSC Xena (https://xenabrowser.net/). Based on this dataset, not just the basic information of ARHGAP44 gene, more importantly, its expression levels, and the broad spectrum of human cancer data including clinical pathological information, cancer subtypes, patient survival status were obtained. The analyses of the association between the ARHGAP44 expression and various clinical traits were conducted using the TCGA pan-cancer data.

### 2.2 Analysis of the basic genetic information and physicochemical properties of the ARHGAP44 gene

Three analysis platforms, namely, ProtParam (online access website: https://web.expasy.org/protparam/), ProtScale (https://web.expasy.org/protscale/), and Human Protein Atlas (https://www.proteinatlas.org/) were accessed to investigate basic information on the ARHGAP44 gene including its genetic and physicochemical properties.

First, ProtParam and ProtScale were applied in succession to analyze the physicochemical properties of ARHGAP44 protein, including amino-acid composition, computed encoding protein molecular weight, theoretical isoelectric point, estimated protein half-life, instability index, hydrophobicity, and hydrophilicity.

Meanwhile, Human Protein Atlas, a reliable tool for protein information annotation, was accessed to predict the cellular location of ARHGAP44, and to preliminarily observe the gene’s expression patterns in normal human tissues and several cancer types.

### 2.3 Expression patterns and association with clinical parameters

Following the characterization of the basic genetic and encoding protein information of ARHGAP44, UALCAN (http://ualcan.path.uab.edu/analysis.html), GEPIA (http://gepia.cancer-pku.cn/), and local hospital samples were jointly used to analyze the expression patterns of ARHGAP44 in human cancers. Online data resources including UALCAN and GEPIA were utilized to analyze ARHGAP44 gene expression in a broad spectrum of human cancers including, but not limited to the following: in the bladder - urothelial carcinoma (BLCA), invasive breast carcinoma (BRCA), cholangiocarcinoma (CHOL), in the kidney - clear cell carcinoma (KIRC) and papillary cell carcinoma (KIRP), in the brain - lower grade glioma (LGG), in the liver - hepatocellular carcinoma (LIHC), in the lung - adenocarcinoma (LUAD), and thymoma (THYM), in comparison with their corresponding normal control tissues.

Furthermore, local hospital samples involving BRCA, KIRC, LUAD, lungsquamous cell carcinoma (LUSC), small cell lung cancer (SCLC), and pancreatic adenocarcinoma (PAAD) were collected to validate ARHGAP44 gene expressions based on an immunochemistry (IHC) experiment, which was conducted following the protocol described below. In addition, the association between the expression and clinical parameters in these cancers was also analyzed.

### 2.4 Immunohistochemistry experiment

#### 2.4.1 Tissue samples and reagents

Cancer samples used for the IHC experiment were obtained from the biobank of the Second Hospital of ShanXi Medical University. All samples were derived from surgical specimens of patients treated at the General Surgery Department and sent for pathology examination at our Pathology Department, and the residual tissues were archived long term in the hospital biobank. Informed consent was obtained from patients by the biobank committee when the tissues were donated. The biobank samples used for this research were approved by the Hospital Institutional Board [The second Hospital of ShanXi Medical University, ShanXi Province, China; approval no. 2023YX(179)].

In the study, 10 cases of BRCA, KIRC, LUAD, LUSC, SCLC, PRAD, and PAAD samples were picked respectively from the biobank after hematoxylin and eosin (H&E) staining confirmation of the disease diagnosis by local hospital pathologists, followed by IHC experiment procedures.

The IHC experiment was performed on sample cancer tissues to preliminarily verify the expression pattern of ARHGAP44 between each type of cancer and matched with paracancerous tissues. The VENTANA platform (Roche, Basel, Swizerland) in a local hospital’s pathology department was used. The primary antibody of ARHGAP44 gene was purchased from ABclonal, Wuhan, China (Catalog no. A7357), and the secondary antibody (Envision/HRP kit) and 3,3’-Diaminobenzidine Tetrahydrochloride (DAB) detection kit were from ZSBG-Bio, Beijing, China. Other reagents including H_2_O_2_, antigen retrieval citrate solution, phosphate-buffered saline (PBS), and hematoxylin stain were all from a local hospital’s supply department.

#### 2.4.2 IHC experimental protocol

Paraffin-embedded cancer tissues were sectioned, deparaffinized, rehydrated, and subjected to antigen retrieval according to the antibody’s manual instruction. Endogenous peroxidase activity was blocked by incubation in methanol containing 0.3% H_2_O_2_ for 10 min, followed by blocking with bovine serum albumin for 30 min. Then, the slides were incubated with primary ARHGAP44 antibody (dilution 1:3,000) overnight at 4 °C, then with secondary antibody for 40 min at 37 °C. Finally, the slides were processed with horseradish peroxidase (HRP) and visualized in DAB for results assessment.

#### 2.4.3 IHC results evaluation

As a cytoskeleton mobility-related gene, ARHGAP44 protein is widely distributed in various tissues. Staining observed in both cell membrane and cytoplasm was regarded as positive IHC staining. IHC results were evaluated by a local hospital’s registered pathologist based on staining intensity and staining area.

The staining intensity was scored with the criteria set as none (0), mild (1), moderate (2), and strong (3). Meanwhile, the staining area was classified as <5% (0), 6%–25% (1), 26%–50% (2), 51%–75% (3), and >75% (4). The final score of each sample equals the multiplication of staining intensity and staining area. With a final score of ≥6, the sample staining would be defined as strongly positive. With a score of 4–6, the result would be defined as moderately positive. Meanwhile, with a score of 1–4, the result would be considered mildly positive. Lastly, if the score is ≤1, the result would be classified as negative.

### 2.5 Quantitative real-time PCR experiments

To preliminarily validate the dysregulated expression of ARHGAP44 gene in cancers, tissue samples of four cancers, namely, LUSC, LUAD, PAAD, and KIRC were selected from the hospital biobank. For each cancer type, mRNA was extracted from 10 paired cancer and adjacent paracancerous tissues for QPCR analysis of the ARHGAP44 gene expression.

Total mRNA was extracted using RNAiso-Plus (TAKARA, DaLian, China). Then, 1 µg extracted mRNA was used for cDNA synthesis with a commercial cDNA synthesis kit (TAKARA, DaLian, China), following the kit’s operating instruction. Furthermore, qPCR was performed on Roche z 480 and the primers used were the following:

ARHGAP44:

Former:ACAACAACATCCGATACTTGA

Reverse: TTGGCATTATGGTTCACG

GAPDH:

Former: AGAAGGCTGGGGCTCATTTG

Reverse: AGGGGCCATCCACAGTCTTC

PCR cycling condition was set as 95 °C for 5 min for 1 cycle; 95 °C for 5 s, 60 °C for 30 s, and 72 °C for 34 s for 35 cycles followed by the melting curve stage. The relative gene expression in each sample was recorded as the average 2^−ΔΔCT calculation result of three replicates. Furthermore, T-test was used for detailed statistical analysis. P<0.05 is considered statistically significant.

### 2.6 Survival analysis of ARHGAP44 gene in human cancers

Survival analysis is essential for evaluating the clinical relevance of dysregulated genes. Kaplan–Meier plotter (http://kmplot.com/analysis/) is a widely used open-access online service containing over 10,000 case samples of human pan-cancer types for analyzing the overall survival (OS) and recurrence-free survival (RFS) of patients. In the study, OS and RFS survival correlation of the ARHGAP44 gene in a broad spectrum of human cancers was evaluated using the Kaplan–Meier plotter platform.

### 2.7 Post-transcription modulation and genetic alterations analysis

To explore the mechanism underlying dysregulated ARHGAP44 expression in various human cancers, we preliminarily analyzed the common post-transcriptional regulatory events, including the methylation and phosphorylation levels of ARHGAP44 gene in cancers using the UALCAN platform (http://ualcan.path.uab.edu/analysis.html), an open-access and user-friendly platform. Researchers only need to choose the “TCGA” data first, then type-in the specific gene name and select the cancer type, and the platform will then automatically generate the analyzed result.

In addition to mRNA dysregulation, other genetic variations for instance gene mutation, copy number variation, amplification, and deletion may change certain gene’s working mode and affect cellular biological functions. cBioPortal (https://www.cbioportal.org/) is one of the largest open-access cancer genomics data website containing over 126 large-scale tumor research projects. It has been working effectively as a rich data resource for worldwide research investigation of variations in certain genes.

In the study, the “cancer types summary” module of the “quick search” section in cBioPortal database was applied to uncover the genetic alteration characteristics of ARHGAP44 in human cancers, while the “mutation” module of the same database was accessed to display the mutated site information of the gene in 3D protein structures.

### 2.8 Protein–protein interaction network construction and related genes analysis

After characterizing the expression and alteration patterns of ARHGAP44 in cancers, we explored its detailed cellular function and potential regulatory mechanisms. To do this, STRING (Search Tool for the Retrieval of Interacting Genes; https://string-db.org/) was used to construct the PPI network centered on the ARHGAP44 gene to investigate its surrounding interacting genes.

Subsequently, gene enrichment analysis was applied to annotate the basic biological attributes of ARHGAP44 and its connecting genes including their main cellular location, associated biological processes, molecular functions, and the signaling pathways in which they are mainly enriched.

### 2.9 Cytoskeleton mobility-related gene expression association analysis

ARHGAP44 is a GAP that regulates RHO GTPases activity, thereby influencing cell mobility and other cytoskeleton-dependent cellular biological processes. To validate the association between the ARHGAP44 gene and cytoskeleton-related genes, 29 genes commonly known to be related with cytoskeleton dynamics—such as actin filament stabilization, F-actin polymerization, and G-actin and F actin equilibrium, and the generation of actin–myosin contractile force—were selected (listed in **Supplementary Table 1**).

Using the TCGA pan-cancer expression data, the correlation between ARHGAP44 and the 29 genes signature was analyzed for preliminary verification of the association between ARHGAP44 and cytoskeleton dynamics in cancers.

### 2.10 Correlation with key Rho GTPase proteins RHOA, RAC1, and CDC42 in cancers

Among identified RHO GTPases, RHOA, RAC1 and CDC42 are the most extensively characterized and regulate distinct aspects of cytoskeleton dynamics. Although previous studies suggest that ARHGAP44 acts as a GAP for RAC1 and CDC42 in certain cancers, its pan-cancer correlation with all three proteins remains incompletely defined.

In the study, using TCGA pan-cancer data, the correlation between the ARHGAP44 gene and each of RHOA, RAC1 as well as CDC42 RHO GTPases proteins was investigated in individual cancer types to improve understanding of the mechanism by which ARHGAP44 regulates RHO GTPases mediated cell mobility.

### 2.11 Extracellular matrix degradation association analysis

Metastasis is a leading cause of cancer-related mortality and involves not only intensive collaboration of cytoskeleton movements, but also changes of the structure of cellular microenvironment, for instance, extracellular matrix (ECM). ECM degradation is commonly known as a major step in cancer metastasis. After analyzing the association between the ARHGAP44 gene and cytoskeleton dynamics, we further explored its relationship with ECM degradation to clarify its potential role in cancer metastasis.

In the study, 23 genes previously known to be related to ECM degradation were selected (listed in **Supplementary Table 2**), and using TCGA pan-cancer expression data, the correlation between ARHGAP44 and the 23 genes signature was analyzed for preliminarily exploring the potential effect ARHGAP44 gene has on ECM structure.

### 2.12 Correlation analysis of cancer stemness, HRD score, and MRR

RNA-based cancer stemness index (RNAss) of each cancer type was calculated based on TCGA pan-cancer data using the “gelnet” R package, followed by investigating its correlation with ARHGAP44 gene expression.

Meanwhile, homologous recombination deficiency (HRD) is an important clinical feature associated with disease progression and the efficiency of PARPi based therapies (30, 31). To further analyze the HRD signature in pan-cancer, 27 commonly adopted genes involved in homologous recombination repair (HRR) signaling pathway (listed in **Supplementary Table 3**) were collected and inputted into GEPIA2.0 to calculate their correlations with ARHGAP44.

In addition to HRD, the association between the ARHGAP44 expression and another well-known DNA deficiency repair system, namely, mismatch repair (MMR)-related proteins were analyzed. Four core MMR proteins, namely, MLH1, PMS2, MSH2, and MSH6 were detected in local hospital cancer samples using the IHC experiment with procedures as described previously, and the primary antibody to all the four MMR proteins were purchased from ZS-BIO (China). The mRNA of corresponding cancer tissues was then extracted for analyzing ARHGAP44 gene expression using QPCR experiment, followed by preliminarily evaluating its association with MMR status.

### 2.13 Cancer proliferation, cell cycle G2M proteins, and EMT association analysis

Uncontrolled cell proliferation is a core hallmark of cancers (32, 33). To comprehensively understand the role of ARHGAP44 gene in cancer development beyond cytoskeleton modulation, the potential connection between the ARHGAP44 expression and cancer proliferation, as well as cell cycle regulation, was explored.

Using the TCGA pan-cancer data, 14 genes commonly known to represent cell proliferation ratio and 199 G2M gene checkpoints related to cell cycle were selected (listed in **Supplementary Table 4**). Moreover, the correlation between the ARHGAP44 expression and the gene clusters was explored.

To further investigate the relevance to cancer epithelial–mesenchymal transition (EMT), another critical step in cancer metastasis, 14 characteristic EMT genes (listed in **Supplementary Table 5**) were selected and inputted into GEPIA2.0 to calculate their correlations with ARHGAP44.

### 2.14 Immune infiltrating cells, immune checkpoints, and tumor mutation burden correlation analysis

To characterize the microenvironment immune landscape in ARHGAP44-high and ARHGAP44-low cancer samples, CIBERSORT algorithm was applied to calculate the relative contents of 22 tumor-infiltrating immune cells (TICs) using the TCGA profile data, followed by analyzing the relationship between the ARHGAP44 gene and the 22 TICs in different types of cancers.

Considering that immune checkpoints, especially CTLA4, PD-L1, TIGIT, TIM-3, and LAG-3, have shown promising drug-targeting effects in multiple cancers and are increasingly accepted as clinical biomarkers for identifying cancer patients most likely to benefit from immunotherapy, we preliminarily investigated the association between ARHGAP44 and the expression of these immune checkpoints.

Furthermore, the association between the ARHGAP44 expression, cytotoxic T lymphocytes (CTLs), and patient survival rates was subsequently assessed using CIDE analysis (https://cide.ccr.cancer.gov), to preliminarily explore the role of ARHGAP44 in regulating the pan-cancer immune microenvironment.

Immune checkpoints, especially PD-L1 expression, MSI, and TMB, have been the three most widely used clinical biomarkers for predicting the potential benefit of immune-targeting therapies to cancer patients. After analyzing the association with immune checkpoint expression, ARHGAP44 gene expression correlation with pan-cancer TMB and MSI scores were evaluated in succession using the ACLBI online database (https://www.aclbi.com/static/index.html#/), an effective and user-friendly data analysis platform.

### 2.15 Drug sensitivity analysis

For the preliminary investigation of the impact of ARHGAP44 on clinical therapeutic responses in human cancers, the ROC Plotter platform (https://www.rocplot.org/) was used. This widely accepted web tool integrates cancer transcriptome data with clinical treatment outcomes to compare gene expression between responders and non-responders and generate therapy-related receiver operating characteristic (ROC) survival curves.

### 2.16 Statistical analysis

Most of the bioinformatics analyses in this study were conducted on specific online platforms using tools available in the platform. For the processing of downloaded TCGA pan-cancer data and local hospital samples QPCR experiment data, all statistical analyses were performed using SPSS 26.0. For the enumeration data, for instance, the analysis of gene expression difference in cancers vs. normal control samples, the data were analyzed using t-test. As for the measurement data such as the association between gene expression and cancer clinical parameters, the data were analyzed by χ2 test. For correlation analysis, for instance, the correlation between gene expression and immune checkpoint expression, the data were analyzed by Spearman analysis. p < 0.05 is considered statistically significant. (For all analysis results, * represents p<0.05, ** represents p<0.01, *** represents p<0.001).

## 3 Results

### 3.1 Analysis of ARHGAP44 genetic and physicochemical properties

Using multiple analytical platforms, the physiochemical properties of ARHGAP44 gene were preliminarily characterized prior to further investigation of its biological functions. The results revealed that ARHGAP44, which is short for Rho GTPase-activating protein 44 and also known as RICH2 or NPC-A-10, is located on 17p12 and contains 22 exons. Meanwhile, the gene encodes 818 amino acids containing protein, and the protein formula was computed as C3917H6283N1087O1216S37 with an estimated weight of 89.2 KD and with a theoretical isoelectric point of 6.13. Moreover, the instability index of the protein is computed to be 71.22 and the grand average of hydrophobic value is -0.461, indicating that ARHGAP44 works as a cellular, unstable, and hydrophilic protein.

Regarding subcellular localization, ARHGAP44 was predicted by Human Protein Atlas to localize in convoluted dendritic plasma membrane sections enriched in polymerized actin and myosin patches, and the gene has been reported to participate in the regulation of plasma membrane-bounded cell projection organization.

### 3.2 ARHGAP44 expression varies in different human cancers

**Figure 1:**
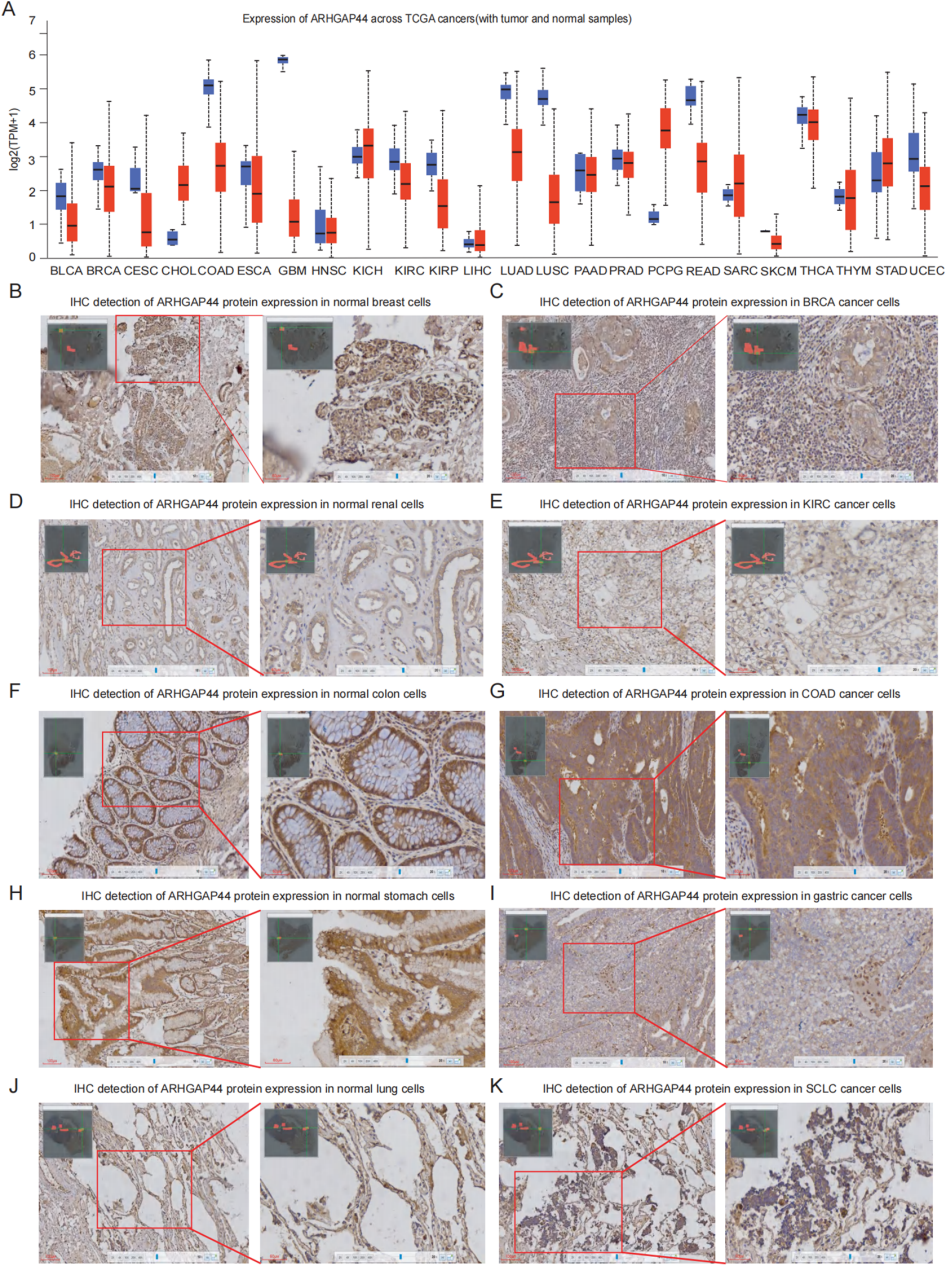
ARHGAP44 gene expression patterns in human cancers. **(A)** UALCAN prediction of ARHGAP44 gene expression in human pan-cancers compared to corresponding normal control samples using the TCGA profiles. IHC experiment detection of ARHGAP44 expression in **(B)** normal breast cells compared to the same case in **(C)** BRCA cancer cells. ARHGAP44 expression in **(D)** normal renal cells compared to same case in **(E)** KIRC cancer cells. ARHGAP44 expression in **(F)** normal colon cells compared to **(G)** COAD cancer cells. ARHGAP44 expression in **(H)** normal stomach cells and corresponding **(I)** gastric cancer cells. ARHGAP44 expression in **(J)** normal lung cells and **(K)** corresponding SCLC cancer cells. (Left graph in each panel: magnification: 100×, error bar represents 100 um. Right graph in each panel: magnification: 200×, error represents 60 um.).

Using the TCGA pan-cancer data, ARHGAP44 mRNA expression in a broad spectrum of human cancers and corresponding normal control samples were investigated, and the results revealed that its expression varies among cancer types. For instance, ARHGAP44 was downregulated in multiple cancers including BLCA, CESC, GBM, KIRP, LGG, LUAD, LUSC, OV, SKCM, and UCEC. In contrast, higher expression was observed in CHOL, DLBC, KICH, PCPG, STAD, and THYM compared with control samples (**Figure 1A**).

ARHGAP44 expression was further validated in several cancer types using local hospital samples. In the study, 10 pairs of samples for BRCA, KIRC, LUAD, LUSC, SCLC, PRAD, and PAAD were used to detect ARHGAP44 expression. Interestingly, IHC-based protein distribution analysis result was partly “inconsistent” with TCGA mRNA analysis result. For instance, in BRCA, the gene protein expression was indicated to be lower in cancer cells with a positivity rate of 30% (3/10) compared to corresponding normal breast cells with a positivity rate of 90% (9/10); the result was consistent with the TCGA result (**Figures 1B, C**). However, as for KIRC, ARHGAP44 protein expression seems similar in cancer cells compared to normal kidney cells with positivity rates of 60% (6/10) and 40% (4/10), respectively, and the weaker staining in cancer cells appears to be because of the specific “transparency” cancer cell morphology (**Figures 1D, E**), which was only partly consistent with the TCGA result. Meanwhile, as for colon adenocarcinoma (COAD), although the gene staining intensity seems weaker in cancer cells compared to colon epithelium cells, the staining area was much wider with positivity rates of 50% (5/10) and 70% (7/10) in cancer and control samples, respectively, which is somehow different from another digestive tract cancer, namely, stomach cancer with both the staining intensity and area weaker in cancer cells compared to normal epithelium cells, and positivity rates were 20% (2/10) and 80% (8/10) in cancer and control samples, respectively (**Figures 1H, J**).

In addition to LUAD and LUSC included in the TCGA data, we tested ARHGAP44 expression in another clinically common type of lung cancer, small cell lung cancer (SCLC). The results revealed that the gene expression was higher in cancer tissues, with a positivity rate of 70% (7/10), compared to normal lung cells, which showed a positivity rate of 40% (4/10) (**Figures 1J, K**).

**Figure 2:**
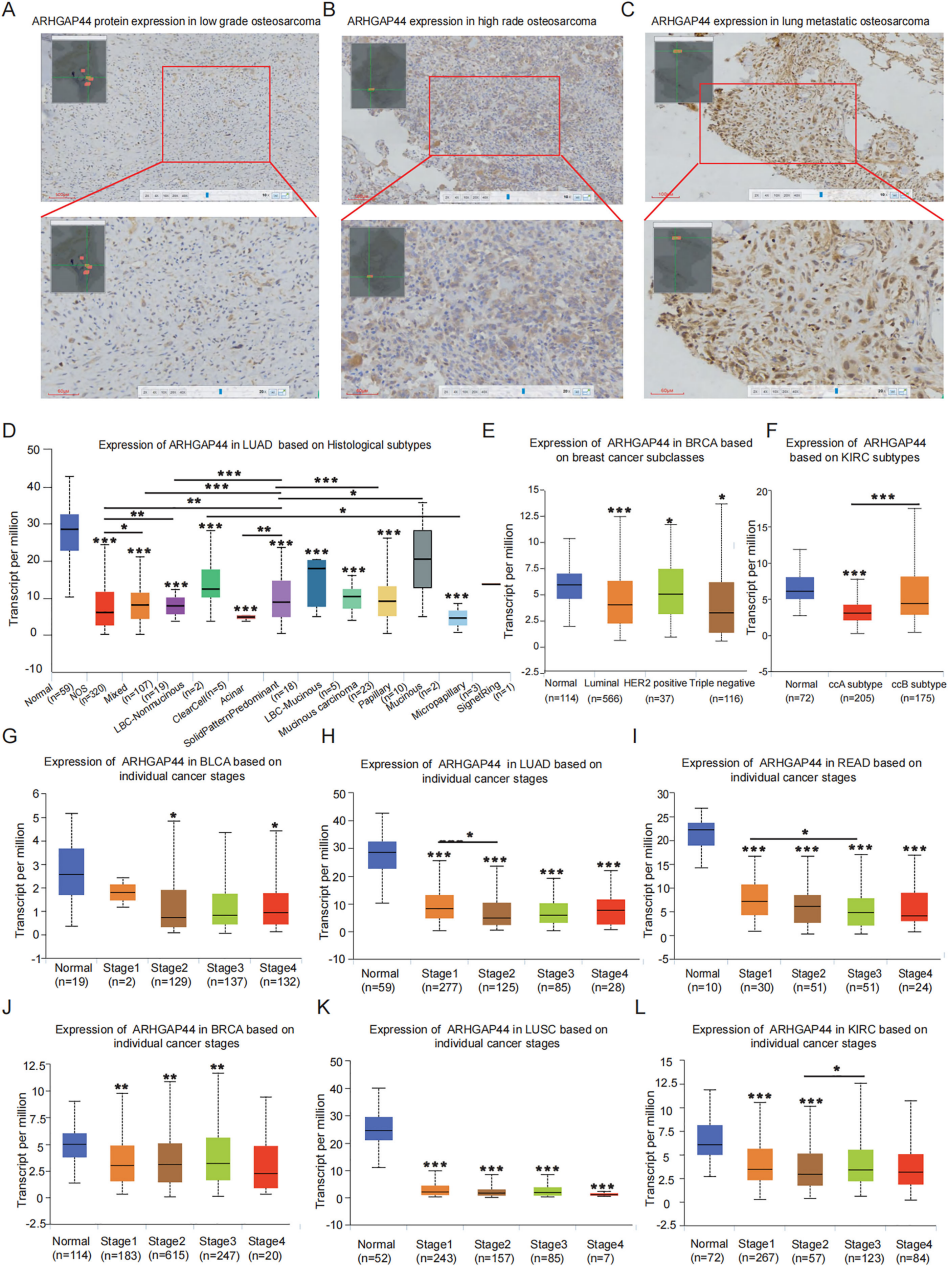
ARHGAP44 gene expression in sarcoma and association with clinical pathological parameters. IHC experiment detection of ARHGAP44 expression in **(A)** low-grade osteosarcoma, **(B)** high-grade osteosarcoma, and **(C)** lung metastatic lesions of osteosarcoma. The various expressions of ARHGAP44 in different subtypes of a single cancer, including in **(D)** LUAD, **(E)** BRCA, and **(F)** KIRC. Expression trends of ARHGAP44 based on the advance cancer stages in **(G)** BLCA, **(H)** LUAD, **(I)** READ, **(J)** BRCA, **(K)** LUSC, and **(L)** KIRC. (*p<0.05, **p<0.01, and ***p<0.001. The first layer * that is right above the error bar represents comparison to normal group, and the above layers *that were above a secondary line represent the comparison between corresponding groups that were covered by the line).

Notably, ARHGAP44 expression also appears to be closely associated with sarcoma progression. We compared the ARHGAP44 protein distribution in 10 low grade, 10 high grade, and 10 lung metastatic osteosarcoma samples, and the results showed progressively elevated ARHGAP44 expression with increasing tumor grade and metastatic potential (**Figures 2A–C**). Although validation in other cancer types remains limited and the sample size is modest, the heterogenetic expression pattern of ARHGAP44 across cancers supports its unique role in human malignancies. The results provide meaningful support for next steps on pan-cancer research of this gene.

### 3.3 ARHGAP44 expression tendency according to clinical parameters in cancer

Analysis of clinicopathological correlations revealed that ARHGAP44 expression not only varies in distinct human cancers but also among histological subtypes within the same cancer, indicating its diverse functions in cancers (**Figures 2D–F**). Regarding other clinical parameters, although ARHGAP44 gene expression tends to decrease as certain cancers progress, for example, in BLCA, LUAD, and READ, especially in early stage I compared to more advanced stages (**Figures 2G–I**), most differences were not statistically significant, for example, as seen in BRCA, LUSC, and KIRC. This suggests that ARHGAP44 does not directly affect cancer stage progression (**Figures 2J–L**). Meanwhile, no specific correlation has been observed between the ARHGAP44 gene and patients’ age, gender, race, smoking and drinking history, or other lifestyle factors related to cancer occurrence (data not shown).

### 3.4 ARHGAP44 affects patient survival in multiple cancers

**Figure 3:**
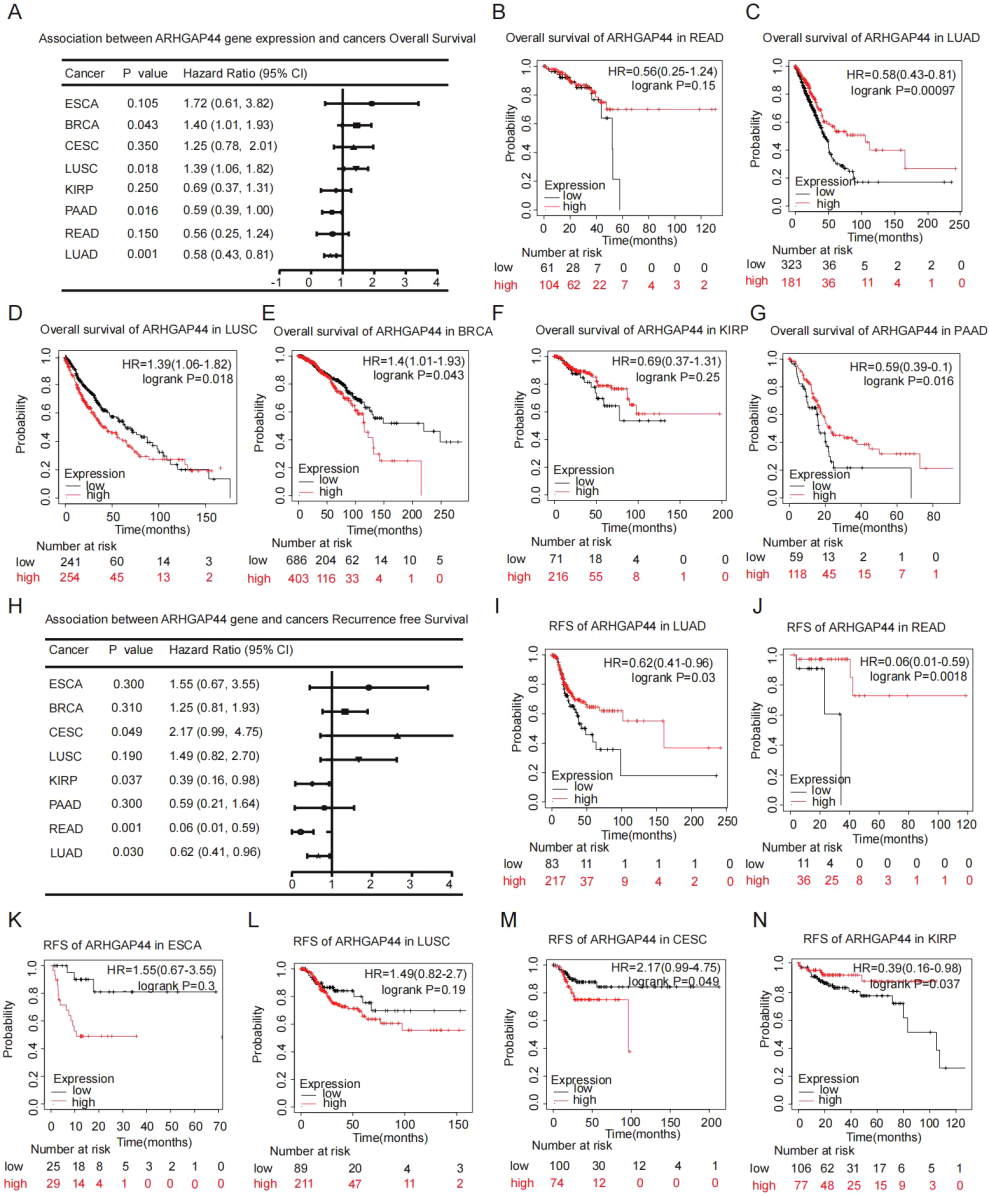
Association between ARHGAP44 and prognostic risk across pan-cancer patients. **(A)** The association between the ARHGAP44 gene and the patients overall survival in different types of cancers. Overall survival analysis of ARHGAP44 in **(B)** READ, **(C)** LUAD, **(D)** LUSC, **(E)** BRCA, **(F)** KIRP, and **(G)** PAAD. **(H)** The association between the ARHGAP44 gene and the patients’ recurrence-free survival in different types of cancers. Recurrence-free survival analysis of ARHGAP44 in **(I)** LUAD, **(J)** READ, **(K)** ESCA, **(L)** LUSC, **(M)** CESC, and **(N)** KIRP. (p<0.05 is considered statistically significant. Only the cancers with statistical significance in OS, RFS, or both are listed).

To evaluate the clinical significance of the dysregulated ARHGAP44 expression in cancers, prognostic association analysis was conducted using the KM plotter platform, and the results revealed that ARHGAP44 gene is associated with patient prognosis in a cancer-dependent manner. We listed the association between the ARHGAP44 expression and the prognosis of several cancers with statistical significance (**Figures 3A, H**). For instance, ARHGAP44 associates with better overall survival (OS) and longer recurrence-free survival (RFS) in LUAD (**Figures 3C, I**), READ (**Figures 3B, J**), PAAD (**Figure 3G**), and KIRP (**Figures 3F, N**), although in some cases, for example, in PAAD, the statistical significance was only in either OS or PFS.

In contrast, high ARHGAP44 expression correlated with worse OS and shorter RFS in BRCA (**Figure 3E**), LUSC (**Figures 3D, L**), CESC (**Figure 3M**), and ESCA (**Figure 3K**). The distinct expression patterns and divergent prognostic value of ARHGAP44 across cancer types indicate its context-dependent functions in cancers originating from different tissues, reflecting both tumor heterogeneity and the complexity of intracellular regulatory networks.

### 3.5 Post-transcription modulation of ARHGAP44 gene in cancers

To explore the mechanism underlying the dysregulated ARHGAP44 expression in human cancers, common post-transcription modulation including the methylation and phosphorylation levels of ARHGAP44 gene in cancers was analyzed. The results revealed that ARHGAP44 methylation level was higher in BRCA, KIRC, COAD, GBM, CESC, and HNSC, which was consistent with the lower gene expression in cancers compared to corresponding normal control samples (**Supplementary Figures 1A–F**). Conversely, the gene methylation level was lower in PCPG and THYM, consistent with the higher expression in cancers vs. normal control (**Supplementary Figures 1G, H**). However, ARHGAP44 methylation level showed inconsistent results in other cancers including CHOL, PRAD, READ, BLCA, THCA, and UCES (**Supplementary Figures 1I–N**), suggesting that DNA methylation contributes only partially to the altered expression of ARHGAP44 in cancers.

**Figure 4:**
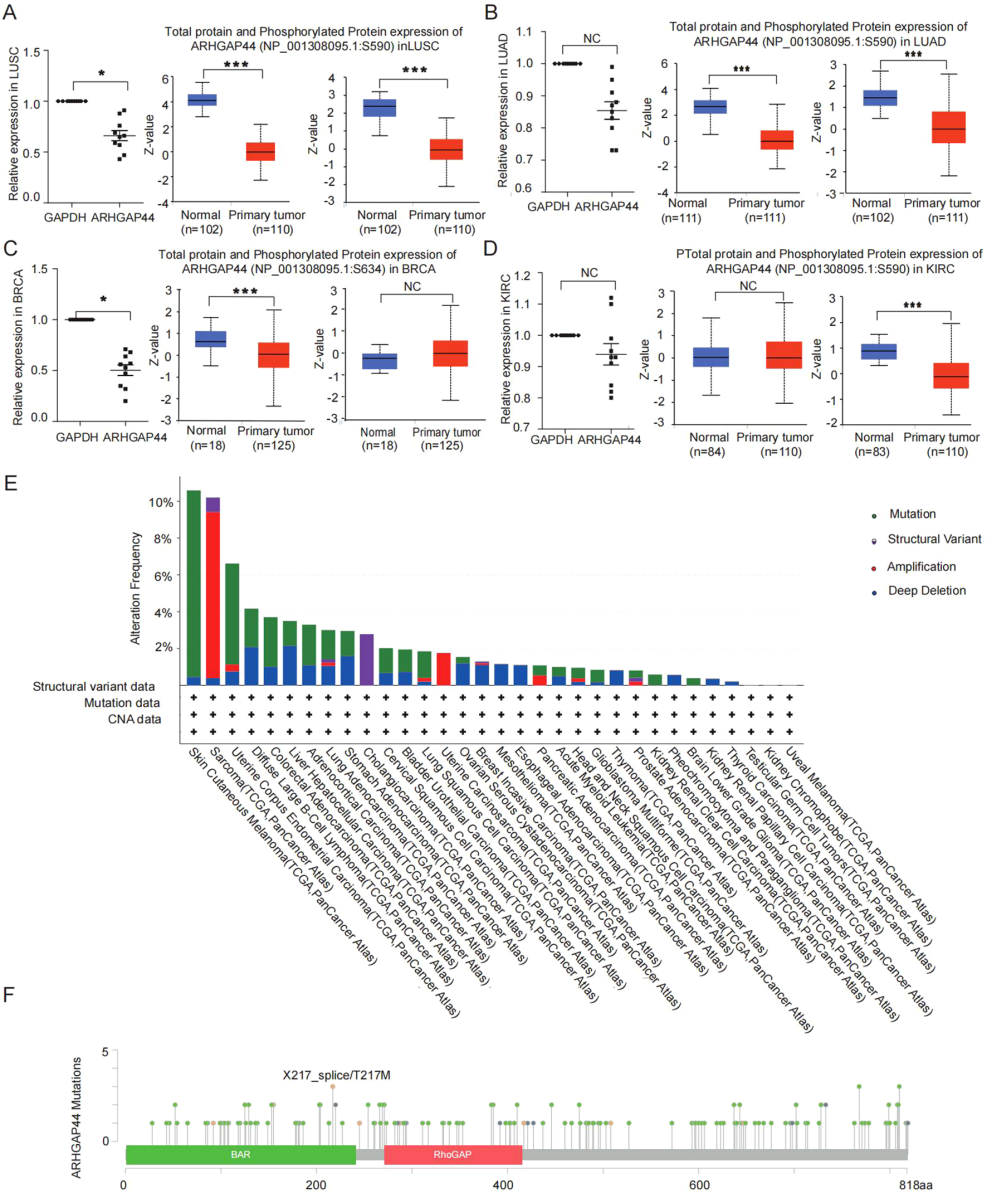
The phosphorylation modulation of ARHGAP44 gene expression and the gene variation types in cancers. The QPCR detection of mRNA expression (left graphic), the total protein expression (middle graphic), and phosphorylated protein expression (right graphic of ARHGAP44 gene in **(A)** LUSC, **(B)** LUAD, **(C)** BRCA, and **(D)** KIRC. The QPCR results were based on 10 pairs of cancer and corresponding normal tissue samples from a local hospital’s biobank, and phosphorylation analysis was performed using the UALCAN platform (*p<0.05, **p<0.01, and ***p<0.001). **(E)** Different types of ARHGAP44 gene variations in human cancers. **(F)** The mutation detection results revealed by cBioPortal dataset.

Meanwhile, a similar trend was observed in the analysis of ARHGAP44 phosphorylation levels. The results indicated that phosphorylated ARHGAP44 protein was consistent with total protein levels in some cancers, including LUAD and LUSC (**Figures 4A, B**), but differed in BRCA and RCC (**Figures 4C, D**). The results also indicate that gene phosphorylation also accounts for only partially altered ARHGAP44 gene expression in cancers. Further deeper analysis is warranted to identify additional regulatory mechanisms of the regulation of ARHGAP44 gene expression in cancers, for instance, potential miRNA or lncRNA targeted regulations.

### 3.6 ARHGAP44 genetic alterations and the association with HRD score in cancers

Apart from mRNA expression, genetic alterations including mutation frequency, protein structure variation, and copy number variation of ARHGAP44 gene were explored using the cBioPortal database. The results revealed that ARGFAP44 alteration profiles vary across tumor types, a certain percent of gene mutation, deletion, and amplification occurs in multiple human tumors (**Figure 4E**). Notably, gene mutations, mostly single nucleotide mutations, were identified in several types of cancers such as skin cutaneous melanoma, uterine endometrioid carcinoma, colorectal adenocarcinoma, lung squamous cell carcinoma, and diffuse large B-cell lymphoma (**Figure 4F**).

**Figure 5:**
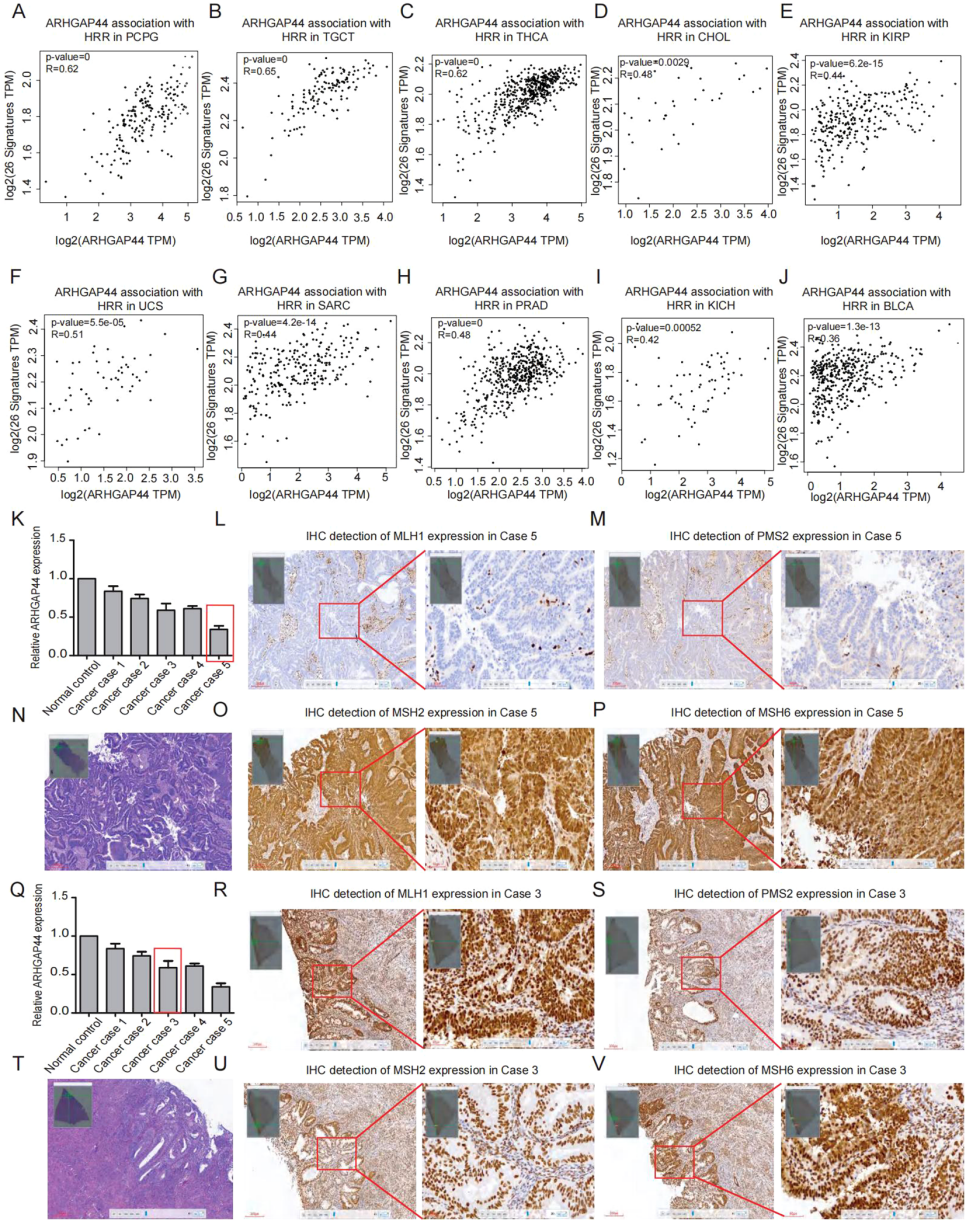
Association between the ARHGAP44 gene and HRR-related gene signature, as well as MRR proteins expression in cancers. The association between the ARHGAP44 gene and HRR gene signature in **(A)** PCPG, **(B)** TGCT, **(C)** THCA, **(D)** CHOL, **(E)** KIRP, **(F)** UCS, **(G)** SARC, **(H)** PRAD, **(I)** KICH, and **(J)** BLCA. (R≥0.30 is considered correlated, an R value between 0.30 and 0.49 is considered preliminarily correlated, and an R value of 0.50–0.69 is moderately correlated, and R≥0.70 is considered strongly correlated). **(K)** QPCR detection of ARHGAP44 expression in five cases of endometrial cancer samples (red box represents the case in L–P). Four MMR proteins, namely, **(L)** MLH1, **(M)** PMS2, **(O)** MSH2, and **(P)** MSH6 in case 5. **(N)** The H&E staining of case 5. **(Q)** QPCR detection of ARHGAP44 expression in five cases of endometrial cancer samples (red box represents the case in R–V). Four MMR proteins, namely, **(R)** MLH1, **(S)** PMS2, **(U)** MSH2, and **(V)** MSH6 in case 3.  **(T)** The H&E staining of case 3.

Homologous recombination repair (HRR) is a major pathway for the resolution of DNA damage and mutations, with key genes including BRCA1, BRCA2, MLH1, MSH2, ATM, FANCA/C, PALB2, RAD51C, and TP53. In the study, we selected 27 commonly known HRR genes and analyzed their correlation with ARHGAP44, and the results revealed that the gene statistically significantly correlates with the 27 genes containing HRR signature in multiple cancers including BLCA, PCPG, TGCT, THCA, CHOL, KIRP, PRAD, KICH, and UCS (**Figures 5A–J**).

Aside from HRD, MRR is another critical DNA deficiency repair system. The association between the ARHGAP44 gene expression and MMR status was preliminarily explored. In the study, we included five cases of endometrial cancer, a common cancer type with high dMMR ratio. Following the IHC experiment of four MMR proteins (MLH1, PMS2, MSH2, and MSH6), one of the five cases was identified as dMMR, showing a loss of MLH1 and PMS2 expression (**Figures 5K–P**), while the remaining four cases were pMMR (**Figures 5Q–V**). Although the analysis was limited by sample size and biobank availability, the lowest ARHGAP44 expression observed in the single dMMR case indicates a potential correlation between this gene and cancer MMR status. Further studies with larger cohorts and more cancer types are needed to validate this correlation.

### 3.7 ARHGAP44 interacting PPI network and related gene enrichment analysis

**Figure 6:**
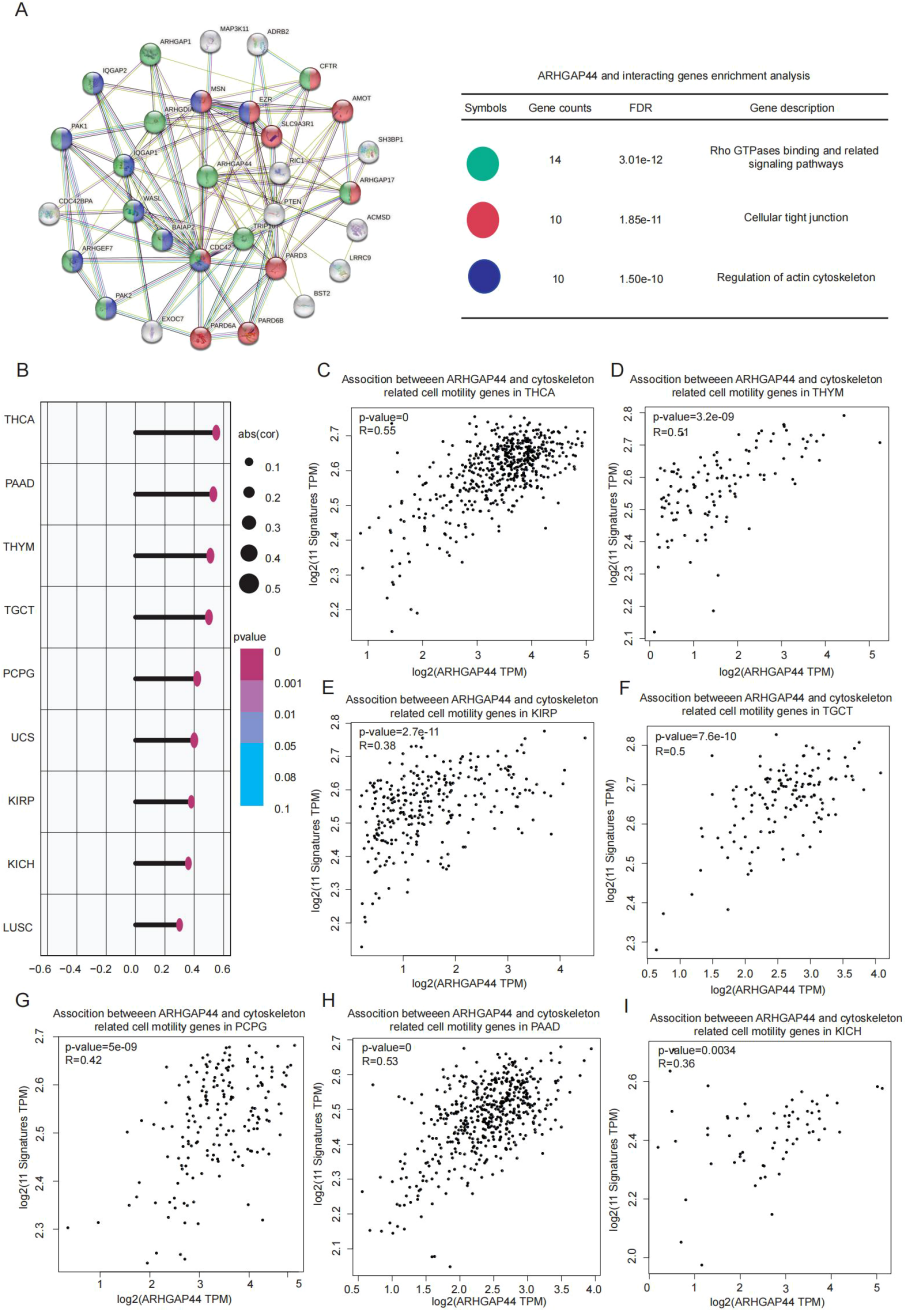
ARHGAP44 interaction and gene enrichment analysis and association with cytoskeleton-related gene signature. **(A)** The PPI network centered on the ARHGAP44 gene (left graphic) for analyzing its main biological functions and its interacting genes (right graphic). **(B)** The association between ARHGAP44 and cytoskeleton-related gene signature in different cancers. ARHGAP44 association with cytoskeleton-related gene signature in **(C)** THCA, **(D)** THYM, **(E)** KIRP, **(F)** TGCT, **(G)** PCPG, **(H)** PAAD, and **(I)** KICH. (R≥0.30 is considered correlated, an R value between 0.30 and 0.49 is considered preliminarily correlated, an R value of 0.50–0.69 is moderately correlated and R≥0.70 is considered strongly correlated).

To explore the detailed biological functions of ARHGAP44 gene and its interacting genes, STRING was used to construct the ARHGAP44 centering PPI network, followed by gene enrichment analysis to reveal the potential signaling pathways in which they are involved. The results were consistent with the current understanding of ARHGAP44 gene as a GAP modulating protein for RHO GTPases. The biological processes mainly enriched among ARHGAP44 and its interacting partners were related to cytoskeleton organization, regulation of actin filaments, and RHO GTPases binding (**Figure 6A**). The results provide robust support for further investigation of the relationship between ARHGAP44 and cytoskeleton-related genes.

### 3.8 ARHGAP44 is associated with multiple cytoskeleton mobility-related genes, especially Rho GTPase proteins RHOA, RAC1, and CDC42 in human cancers

To evaluate the correlation between ARHGAP44 and cytoskeleton-related genes across human cancers, we selected 29 genes reported to participate in cytoskeleton dynamics, including actin filament stabilization, F-actin polymerization, and actin–myosin contractile force generation. The result showed that ARHGAP44 was positively correlated with the 29 genes containing signature in at least nine cancers, namely, THCA, PRAD, THYM, TGCT, KICH, KIRP, PCPG, LUSC, and UCS (**Figures 6B–I**).

**Figure 7:**
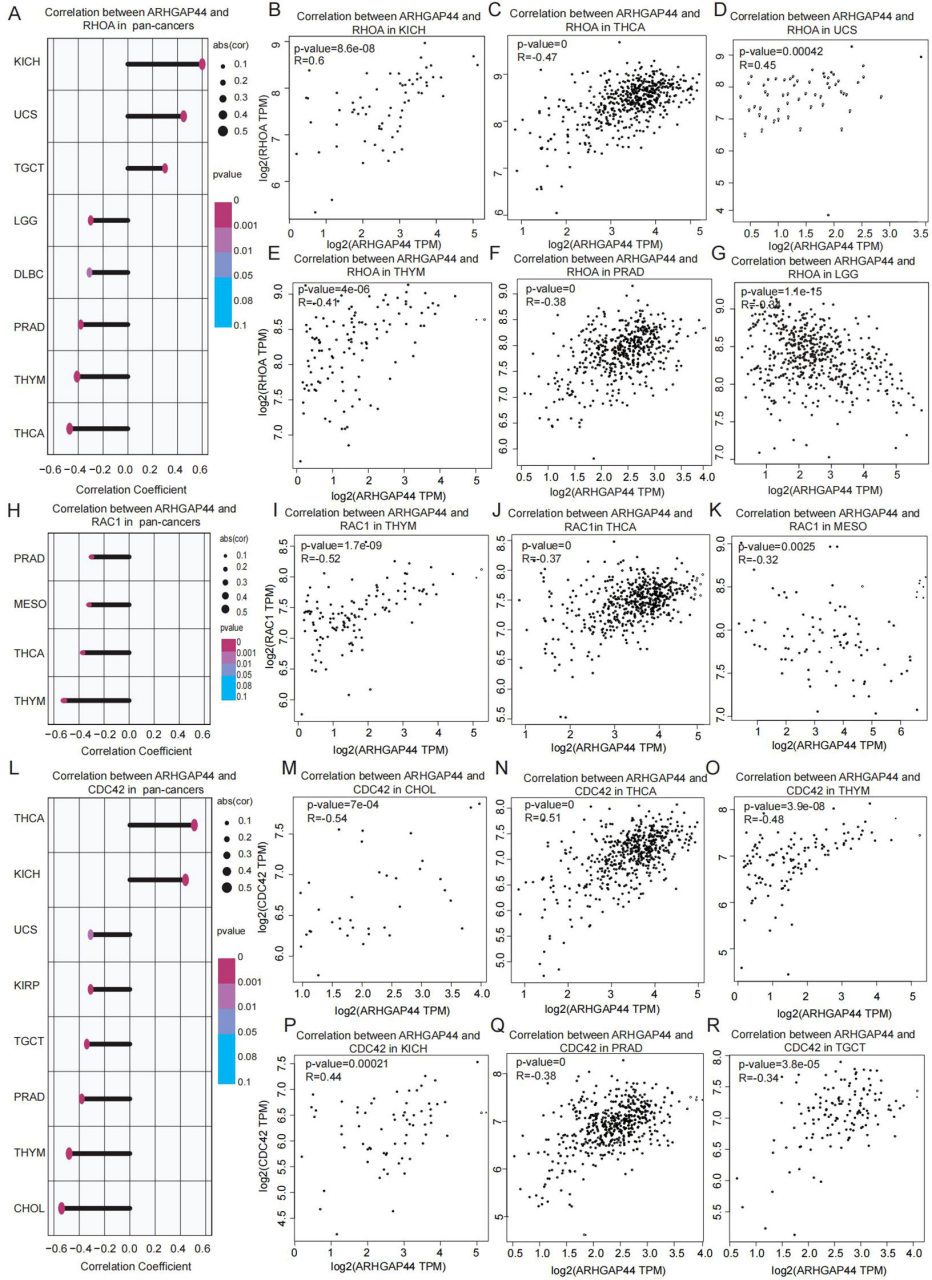
Association between ARHGAP44 and three main Rho GTPases in cancers. **(A)** ARHGAP44 association with Rho GTPase RHOA in different cancers, including **(B)** KICH, **(C)** THCA, **(D)** UCS, **(E)** THYM, **(F)** PRAD, and **(G)** LGG. **(H)** ARHGAP44 association with Rho GTPase RAC1 in multiple cancers, including in **(I)** THYM, **(J)** THCA, and **(K)** MESO. **(L)** ARHGAP44 association with Rho GTPase CDC42 in different cancers, including in **(M)** CHOL, **(N)** THCA, **(O)** THYM, **(P)** KICH, **(Q)** PRAD, and **(R)** TGCT. (Only cancers with R>0.3 are listed. R≥0.30 is considered correlated, an R value between 0.30 and 0.49 is considered preliminarily correlated, an R value of 0.50–0.69 is moderately correlated, and R≥0.70 is considered strongly correlated).

Considering that RHOA, RAC1, and CDC42 are the three best characterized RHO GTPases, we further examined their correlation with ARHGAP44 at the pan-cancer level. The results revealed that ARHGAP44 was associated with RHOA in KICH, LGG, UCS, TGCT, THCA, THYM, PRAD, and DLBC (**Figures 7A–G**), and with RAC1 in MESO, PRAD, THCA, and THYM (**Figures 7H–K**). Meanwhile, the correlation with CDC42 was discovered in CHOL, KICH, KIRP, UCS, TGCT, THCA, THYM, and PRAD (**Figures 7L–R**).

A notable phenomenon worthy of further investigation was that ARHGAP44 was found to correlate with all three RHO GTPases (RHOA, RAC1, and CDC42) in three cancers, namely, PRAD, THCA, and THYM, suggesting a coordinated regulatory network through which ARHGAP44 modulates Rho GTPase-dependent cytoskeleton dynamics in these cancers. The phenomenon provides a promising direction for further clinical studies on cell migration and cytoskeleton-mediated cancer metastasis.

### 3.9 ARHGAP44 correlates with ECM degradation and EMT process in cancer metastasis

In addition to the intensive collaboration of cytoskeleton movements, remodeling of the cellular microenvironment structure particularly ECM degradation, represents a crucial step in cancer cell migration and metastasis. We therefore analyzed the association between ARHGAP44 and a signature composed of 23 ECM degradation-related genes. The results showed a significant correlation was discovered in eight cancers, namely, THYM, HNSC, KIRC, KIRP, CHOL, SKCM, THCA, and LIHC (**Supplementary Figures 2A–G**). Interestingly, THYM and THCA, which previously showed positive correlations between RHO GTPase-related cytoskeleton dynamics and ARHGAP44 gene, also exhibited consistent associations with ECM degradation, further supporting the involvement of ARHGAP44 in cancer metastasis.

Furthermore, significant correlations between the ARHGAP44 expression and EMT signature were detected in CHOL, KIRC, LIHC and THYM (**Supplementary Figures 2H–N**), the correlation with multiple critical clinical cancer traits indicating ARHGAP44 works as a potential biomarker for cancer progression.

### 3.10 ARHGAP44 correlates with multiple cancer stemness index

**Figure 8:**
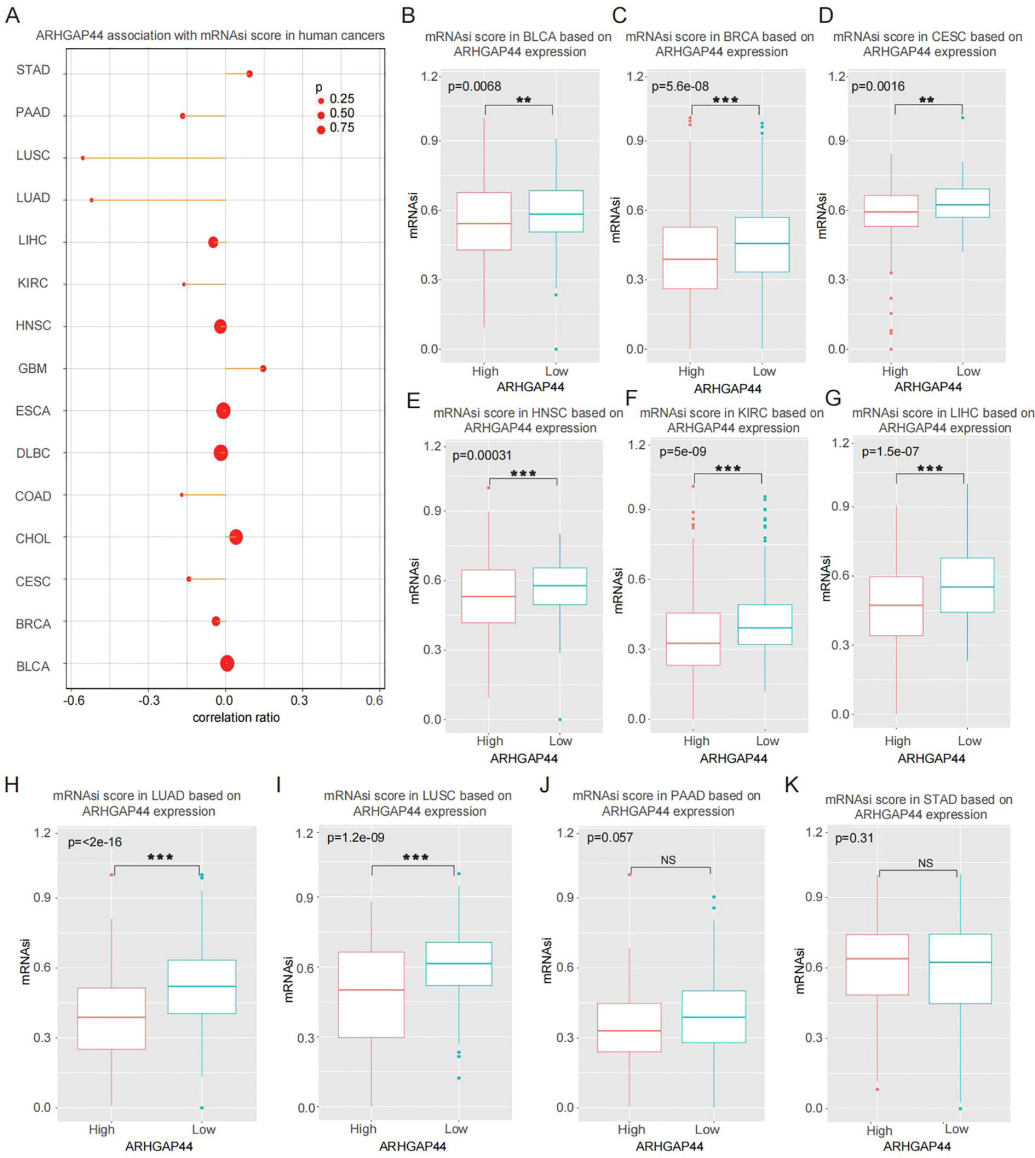
Cancers mRNAsi score distribution in different ARHGAP44 gene expressed samples. **(A)** ARHGAP44 association with mRNAsi score in different cancers. Comparison of mRNAsi score in high-ARHGAP44 and low expression groups in **(B)** BLCA, **(C)** BRCA, **(D)** CESC, **(E)** HNSC, **(F)** KIRC, **(G)** LIHC, **(H)** LUAD, **(I)** LUSC, **(J)** PAAD, and **(K)** STAD. (*p<0.05, **p<0.01, and ***p<0.001).

Cancer stemness index is an indicator that evaluates the similarity between tumor cells and stem cells, which is related to the active biological processes in cancer cells as well as the lower degree of tumor differentiation (34–36). Using the TCGA pan-cancer gene expression data, we calculated the mRNA expression-based stemness index (mRNAsi) in each cancer and evaluated its correlation with ARHGAP44 gene expression (**Figure 8A**). The result revealed that the mRNAsi score differed significantly between the high-ARHGAP44 expression and low-expression groups in multiple cancers including BLCA, BRCA, CESC, HNSC, KIRC, LIHC, LUAD, and LUSC (**Figures 8B–I**); however, in some cancers such as PAAD and STAD, the score difference was not statistically significant (**Figures 8J, K**).

### 3.11 ARHGAP44 negatively correlates with cancer proliferation and cell cycle progression

To further characterize the function of ARHGAP44 in key cancer-related phenotypes, we analyzed the association between the gene expression and 14 cancer proliferation-related genes and 199 genes representative of the G2M cell cycle checkpoint. The results revealed that ARHGAP44 expression was negatively correlated with both cancer proliferation (**Supplementary Figures 3A–H**) and cell cycle progression (**Supplementary Figures 3I–M**) in ACC, LGG, LUAD, MESO, OV, PAAD, THYM, and TGCT, highlighting the functional diversity of ARHGAP44 across human cancers.

### 3.12 ARHGAP44 is involved in the immune infiltration landscape and immune checkpoint expression in multiple cancers

**Figure 9:**
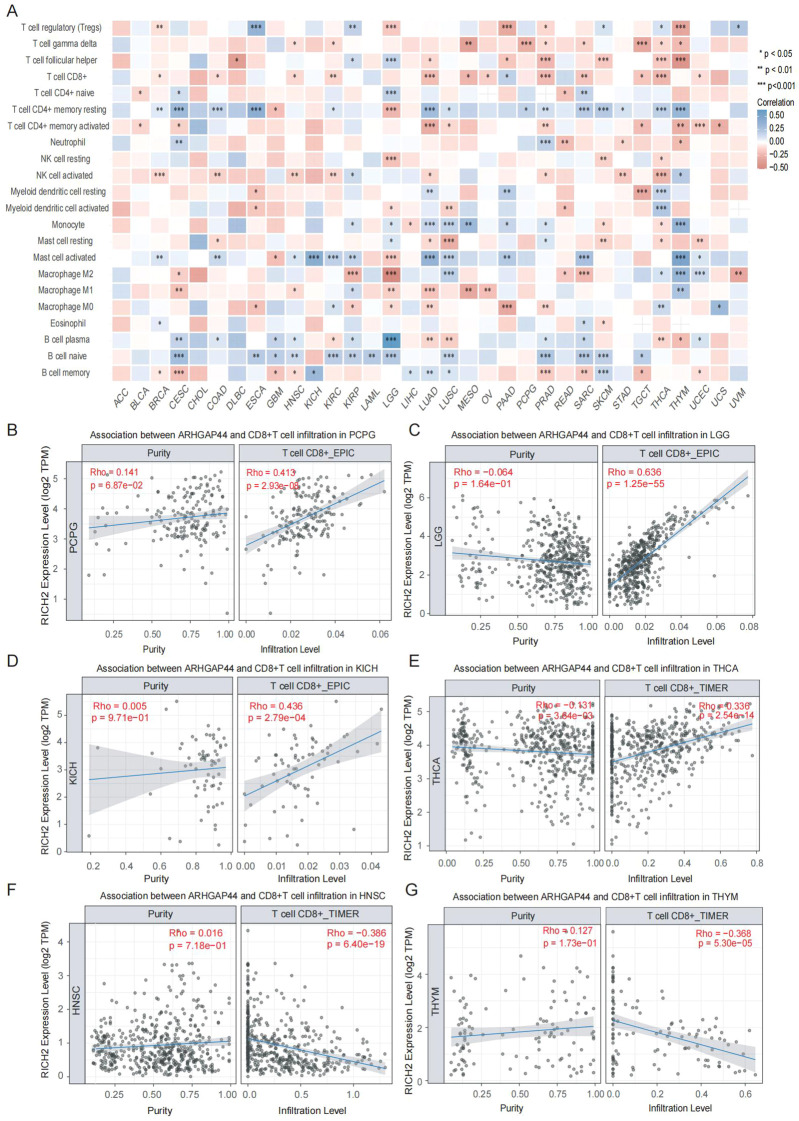
ARHGAP44 association with TIC distribution and CD8+ T-cell infiltration in cancers. **(A)** Association between the ARHGAP44 gene and 22 TICs distribution in pan-cancer. Association between ARHGAP44 and CD8+ T-cell infiltration in **(B)** PCPG, **(C)** LGG, **(D)** KICH, **(E)** THCA, **(F)** HNSC, and **(G)** THYM. (An R value between 0.30 and 0.59 is considered preliminarily correlated, an R value of 0.60–0.79 is moderately correlated, and R>0.80 is considered strongly correlated). (*p<0.05, **p<0.01, and ***p<0.001).

To investigate the immunological role of ARHGAP44 in the tumor microenvironment, we first used the CIBERSORT algorithm to evaluate correlations between ARHGAP44 and 22 tumor-infiltrating immune cell (TIC) subsets. As shown in the heatmap, strong positive correlations were observed between ARHGAP44 and multiple TICs including T-cell CD4+ memory cells, monocytes, activated mast cells, and naive B cells in several cancers. Conversely, negative correlations were detected with T-helper cells, M0 macrophages and M1 macrophages in other cancer types (**Figure 9A**).

Given that cytotoxic T lymphocytes (CTL) are the major affected cells during tumors immunosuppression, the association between ARHGAP44 and CD8+ T-cell distribution was evaluated next. The result indicated that ARHGAP44 was significantly positively correlated with immune cell infiltration in multiple cancers, for instance, in PCPG, LGG, KICH, and THCA (**Figures 9B, E**). However, in other cancers, such as HNSC and THYM, negative correlations were discovered, indicating the cancer-independent effect ARHGAP44 has on immune cell infiltration (**Figures 9F, G**).

We also investigated ARHGAP44 association with CTL functions following TIDE analysis. The results revealed that CTL dysfunction levels significantly differed between high-ARHGAP44 expression and low-expression samples in multiple cancers including LUAD, LUSC, SKCM, BLCA, KIRP, BRCA, and KIRC. These findings highlight the cancer-specific impact of ARHGAP44 gene on CTL function (**Supplementary Figures 4A–G**).

In addition to immune cell infiltration, the association between the ARHGAP44 gene and promising clinical immune checkpoints including PD-L1, CTLA4, TIGIT, TIM-3, and LAG-3 were evaluated. Significant associations were detected across multiple cancer types. Notably, ARHGAP44 showed opposite correlations with the same immune checkpoint in different cancer types. For example, ARHGAP44 was positively correlated with CTLA4 in STAD, SKCM, and LIHC, but negatively correlated in OV, MESO, LUAD, and LGG, demonstrating its context-dependent immunomodulatory functions (**Supplementary Figure 4H**).

### 3.13 Tumor mutation burden and microsatellite instability analysis

**Figure 10:**
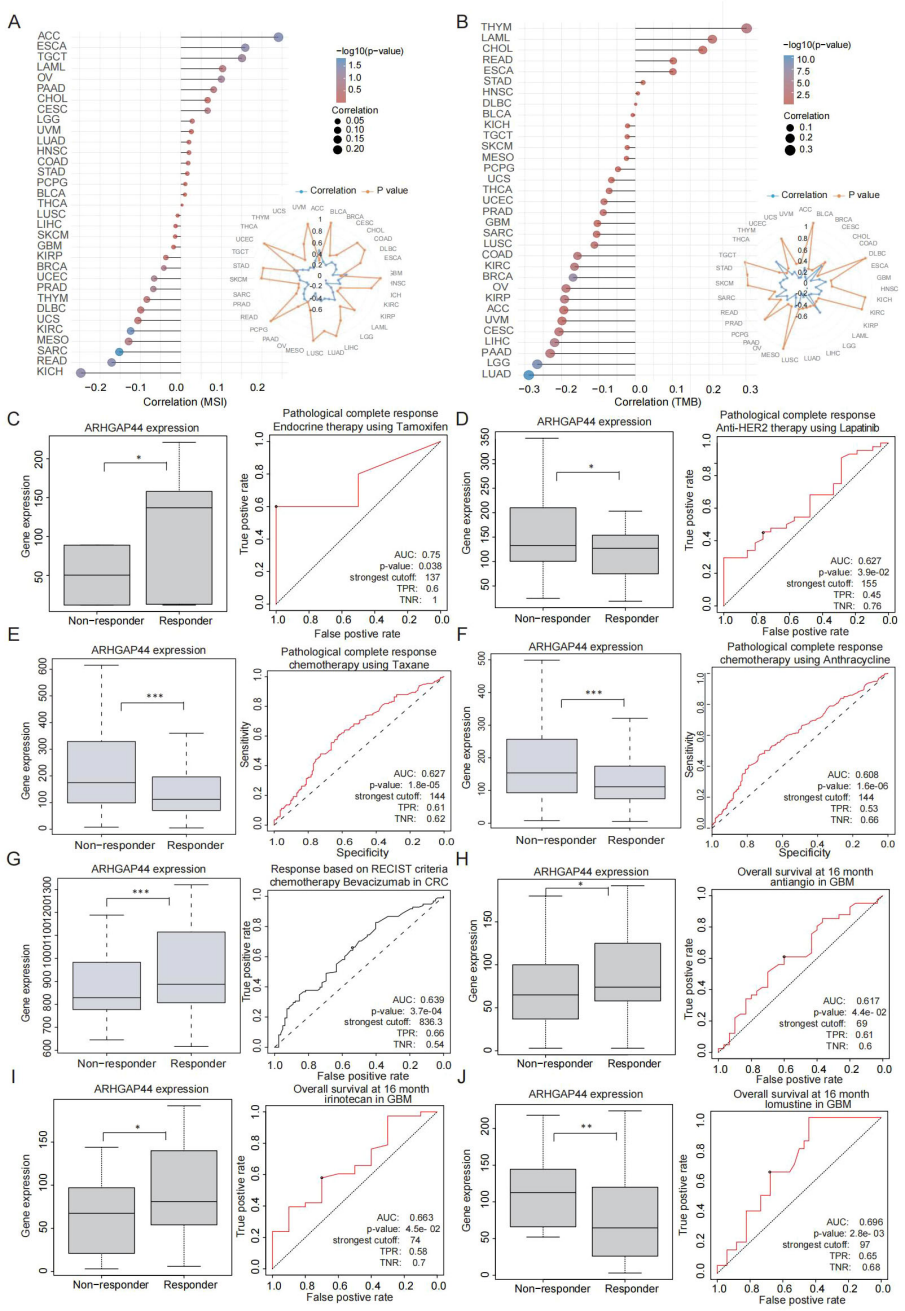
ARHGAP44 association with cancers MSI and TMB as well as certain drug sensitivity. ARHGAP44 association with tumor **(A)** MSI and **(B)** TMB status in cancers. ARHGAP44 expression difference between breast cancer responders and non-responder patients as well as the generated AUC curve after using **(C)** endocrine therapy tamoxifen, **(D)** anti-Her-2 therapy lapatinib, **(E)** chemotherapy taxane, and **(F)** anthracycline. ARHGAP44 gene expression difference between colorectal cancer responders and non-responder patients as well as the generated AUC curve after using **(G)** bevacizumab. ARHGAP44 gene expression difference between GBM responders and non-responder patients as well as the generated AUC curve after using **(H)** antiangio, **(I)** irinotecan, and **(J)** lomustine. (*p<0.05, **p<0.01, and ***p<0.001).

Tumor mutation burden (TMB) and microsatellite instability (MSI) are important clinical features of the tumor immune microenvironment and serve as useful biomarkers for predicting responses to immune-targeted therapies (37). Their status directly influences clinical immunotherapy strategies. ARHGAP44 expression showed a moderate positive correlation with MSI in ACC and a moderate negative correlation in KICH (**Figure 10A**). Meanwhile, a positive correlation was found between the ARHGAP44 expression and TMB in THYM, LAML, and CHOL, and the association turned to be negative in LUAD, LGG, PAAD, and some other cancer types (**Figure 10B**).

### 3.14 ARHGAP44 affects drug sensitivity in certain therapies

Drug sensitivity remains a critical and often an insurmountable problem in clinical cancer treatment. To assess whether ARHGAP44 could predict therapeutic responses, we analyzed clinical treatment data from ROCplotter to evaluate the association between therapeutic outcomes and ARHGAP44 expression in selected cancer types. In BRCA, ARHGAP44 expression was significantly higher in responders than in non-responders to tamoxifen endocrine therapy (**Figure 10C**); however, gene expression was lower in responders to lapatinib anti-HER2 therapy (**Figure 10D**) and taxane- or anthracycline-based chemotherapy (**Figures 10E, F**).

Meanwhile, in colorectal cancer, ARHGAP44 gene expression was higher in responders compared to non-responders after receiving the chemotherapy drug bevacizumab (**Figure 10G**). In GBM, responders to antiangiogenic therapy and irinotecan showed higher ARHGAP44 expression compared with non-responders (**Figures 10H, I**), whereas lower expression was observed in responders to lomustine (**Figure 10J**). The area under the curve (AUC) values for the above ranged from 0.61 to 0.75, supporting the reliability of the results.

Additionally, using the RNAactDrug platform, the top 10 compounds correlated to ARHGAP44 mRNA expression with FDR < 0.05 are given in **Table 1**. Although further experimental validation and clinical trials are required before clinical application, the results provide valuable insights for future drug development and targeted therapy research.

**Table 1:** The 10 compounds correlated with ARHGAP44 using the RNAactDrug platform.

Compounds	Omics	Source	Spearman.stat	Spearman.fdr
sri1215	Methylation	CellMiner	0.534	0.0002
riboprine	Methylation	CellMiner	0.422	0.0252
simeon	Methylation	CellMiner	0.415	0.0304
1,4-dimethoxy-7-azaisoindole[2,1-a]quinoxalin-6(5h)-one	Methylation	CellMiner	0.413	0.0272
8-hydroxy-3-phenylisocoumarin	Expression	CellMiner	0.405	0.0444
eupalmerin acetate	Methylation	CellMiner	0.404	0.0382
purpureagitosid	Methylation	CellMiner	0.403	0.0453
2’-deoxy-2’-fluorocytidine	Methylation	CellMiner	0.403	0.0446
sb-682330-a	Methylation	CellMiner	-0.411	0.0079
clomid	Methylation	CellMiner	-0.416	0.0143

## 4 Discussion

Cancer is one of the major global health threats, and its initiation involves multiple steps including the accumulation of genetic mutations and alterations that promote the transition of normal cells to a progressive neoplastic state. Over recent decades, the 10 hallmarks of cancers have been widely recognized including uncontrolled cell proliferation, evasion of growth suppressors, resistance to cell death, genome instability and mutation, induction of angiogenesis, replicative immortality, and more threateningly, activation of invasion and metastasis, which is a major cause of cancer-related mortality, as well as reprogramming of cellular energy metabolism, evasion of immune surveillance for destruction, and tumor-promoting inflammation (33).

Although our understanding of cancer has advanced considerably over the past decades, including improved insights into interactions between cancer cells and tumor microenvironment, epidemiological risk factors for carcinogenesis, and genetic alterations, especially “driver” genes that not only initiate cancer development but also serve as potential therapeutic targets, cancer remains challenging. Current knowledge of cancer is still insufficient compared to the highly heterogeneous, complicated, and progressive nature of cancer. Therefore, it is of critical significance to continue investigating collaborative genetic networks in cancers and to identify potential disease-causing gene alterations as well as promising drug targets.

Rho GTPases are a family of small G proteins that contribute to nearly all hallmarks of cancer, including the maintenance of cell morphology and polarity, regulation of cytoskeleton dynamics and cell motility, modulation of cell growth and cell cycle progression, as well as the effects on stem cell differentiation, cell survival, and new gene expression. Although Rho GTPases have emerged as novel therapeutic targets, their roles in different cancer types remain controversial (12). Initially, Rho GTPase family proteins have been proposed to activate cancer formation and progression. The best characterized members RhoA, Rac1, and Cdc42 have been shown to be critical for cell migration by regulating cell contraction and membrane protrusion, suggesting a crucial role in cancer cell invasion and metastasis (38, 39).

However, while several Rho GTPases are indeed upregulated in human cancers, activating mutations of Rho GTPases appear to be rare. Furthermore, mouse models have revealed that deletion of certain Rho GTPase genes does not promote a general tumor growth effect (40–42). The inconsistent findings from cell lines, animal models, and clinical samples imply complex and context-dependent functions of Rho GTPases and their regulatory proteins across human cancers. A comprehensive pan-cancer analysis integrating multidimensional biological features will help clarify the roles of such genes in cancer development.

In this study, we focused on ARHGAP44, a GAP protein of Rho GTPases. ARHGAP44 was initially identified in our previous studies as a recurrent differentially expressed gene in primary tumors compared to matched normal tissues across several cancer types (43–45). To date, only limited functions have been reported about the gene. Considering its potential biological and clinical relevance, TCGA pan-cancer profiles and various bioinformatics-analyzing tools were combined and used to preliminarily explore the roles of ARHGAP44 gene in cancers.

Following a basic understanding of the physiochemical properties of the ARHGAP44 gene, which suggest that ARHGAP44 functions as a cellular, unstable, and hydrophilic protein, its gene expression patterns and their correlation with prognosis were comprehensively investigated. We observed that gene expression varies among different cancer types, notably, an inconsistent mRNA expression and protein distribution trend were observed. For instance, ARHGAP44 mRNA was downregulated in multiple cancers including BLCA, CESC, GBM, KIRP, LGG, LUAD, LUSC, OV, SKCM, and UCEC. Interestingly, in LUAD and RIRP, high transcription of ARHGAP44 was associated with better OS and longer RFS, although in BLCA, LUSC, and CESC, the gene was related with worse OS and RFS survival. The seemingly “inconsistent” mRNA and protein expression trend as well as the associated different survival rates suggest complex regulatory mechanisms governing ARHGAP44 expression in a tissue-dependent manner. A gene that is upregulated in cancer compared to normal tissues does not necessary mean that it is also highly expressed in more advanced cancers, and also, a gene’s gain/loss of transcription or expression in cancer versus normal samples does not necessarily equal to a tumor promoter/inhibitor role. The functions and roles of genes should be highly specific in different types of cancers.

To explore the mechanisms underlying dysregulated ARHGAP44 expression, common post-transcription modulation including methylation and phosphorylation levels of ARHGAP44 gene in cancers was explored. The result supported that gene methylation and phosphorylation account for at least part of the altered expression of ARHGAP44 in cancers. Meanwhile, given that other types of genetic alterations including mutation ratio, protein structural variants, and copy number variations also affect gene cellular activity and function, we analyzed the ARHGAP44 gene alterations across cancers. We found that a certain percentage of gene mutations, mostly single-nucleotide variants, as well as deletions and amplifications, were present in several cancer types including SKCM, LUSC, and READ. Importantly, ARHGAP44 was not only occasionally mutated in cancers but also associated with the cellular DNA repair machinery.

The DNA repair system is an important cellular defense system that corrects DNA replication errors and damages induced by external stimuli such as environmental toxins, UV light, radiation therapy, and chemotherapy (46, 47). Major DNA repair systems include base excision repair (BER), nucleotide excision repair (NER), mismatch repair (MRR), homologous recombination repair (HRR), and non-homologous end joining (NHEJ) (48, 49). Upon activation, repair proteins are recruited to the nucleus to resolve DNA lesions, thereby arresting cells in the S, G2, and M phases of the cell cycle. In this study, ARHGAP44 expression was correlated with MSI and HRR systems in several cancers including BLCA, PCPG, TGCT, THCA, CHOL, KIRP, PRAD, and KICH. ARHGAP44 was also negatively associated with cell proliferation and G2M checkpoint-related cell cycle progression in multiple cancers. These findings support that ARHGAP44 may act as a key component of DNA repair system and contribute to the maintenance of cancer stemness.

As a known GAP for modulating the activity of RHO GTPases, we further analyzed the correlation between ARHGAP44 and the three major Rho GTPases, namely, RHOA, RAC1, and CDC42, across cancers. The results revealed that ARHGAP44 was associated with distinct subtypes of these three Rho GTPases in different cancer types. Notably, ARHGAP44 was correlated with all three Rho GTPases in some cancers including PRAD, THCA, and THYM, indicating the complex but collaborative network of ARHGAP44 regulation on cancer cytoskeleton dynamics. Although compared to gene expression, it would be more meaningful to evaluate the GTP- or GDP-bound state of the RHO GTPases and identify the effect ARHGAP44 gene has on the state transition of the genes, which would require extensively designed cell line experiments. Current results shall provide a useful direction for further experiments and research design.

In addition to its involvement in cancer metastasis via cytoskeleton-related genes and ECM degradation, the potential effect ARHGAP44 has on cancer microenvironment modulation, more specifically, the immune infiltration landscape was explored. Tumor microenvironment was able to exert great influence on tumor proliferation, metastasis, and even environment angiogenesis, especially, evasion of immune surveillance and tumor-promoting inflammation have been well accepted cancer hallmarks. However, the function of ARHGAP44 in tumor immune environment has not been previously elucidated. Our results demonstrated that ARHGAP44 was strongly correlated with multiple TICs. for instance, T-cell CD4+ memory cells, monocytes, activated mast cells, and naive B cells in multiple cancers, and also, CTL dysfunction levels significantly differ between high-ARHGAP44 expression and low expression samples. The results suggest that ARHGAP44 may actively regulate tumor-immune microenvironment.

To preliminarily assess the potential of ARHGAP44 as a therapeutic target, we investigated its association with patient response to certain clinical therapies, and discovered that ARHGAP44 expression level differed significantly between responders and non-responders to several therapeutic agents. Although further *in vitro* validation and clinical trials are required before clinical application, the results provide promising directions for further translational research.

In conclusion, our study reveals the complex and multifaceted roles of ARHGAP44 in cancer progression and clinical outcomes. ARHGAP44 expression varies across cancer types and is associated with diverse patient prognoses, with high expression predicting favorable survival in some cancers but poor outcomes in others. Functionally, ARHGAP44 is correlated with diverse cancer-related processes including EMT, ECM degradation, and immune cell infiltration. It also participates in the DNA repair system including MMR and HRR, and contributes to genome stability and cancer stemness in specific tumor types. Notably, ARHGAP44 is involved in both cytoskeleton-driven metastasis and cancer immunity. Its function is highly dependent on the Rho GTPase profile of the tissue of origin. These findings enhance our understanding of the molecular mechanisms underlying ARHGAP44-mediated regulation of cancer development. Further mechanistic and clinical studies are warranted to validate these observations and promote the clinical utility of ARHGAP44 as a prognostic biomarker and therapeutic target.

## Data Availability

TCGA pan-cancer profiles used in the study can be downloaded from UCSC Xena (https://www.cancer.gov/ccg/research/genome-sequencing/tcga). The original contributions presented in the study are included in the article and **Supplementary Material**. Further inquiries can be directed to the corresponding authors.
