# Supplementary material for: ARHGAP44 gene: a cytoskeleton mobility-related modulator with implications in pan-cancer prognostic risk and immune regulation
Source: Front Oncol. 2026 Mar 31;16:1721943. doi: 10.3389/fonc.2026.1721943 (PMC13076174; doi:10.3389/fonc.2026.1721943)
Supplement: Supplementary file 1 [file DataSheet1.docx]

Supplementary Material

# Supplementary Tables

# 1.1 Supplementary **Table 1. The 29 cytoskeleton dynamics related genes for evaluating the association with ARHGAP44 gene**

| Detailed list of the 29 selected cytoskeleton dynamics relating genes | | | | |
| --- | --- | --- | --- | --- |
| RHOA | CDC42 | RAC1 | PI3-K | ROCK |
| WASP | ARP2 | ARP3 | MRCK | MLC |
| MLCP | IQGAP | b-Catenin | PAK | LIMK |
| Cofilin | Adducin | ERM | mDia | profilin |
| FMNL2 | α-actinin | myosinIIA | paxillin | Fascin |
| Vinculin | Talin | JNK | Cortactin |  |

# 1.2 Supplementary Table 2. The 23 selected genes for representing ECM degradation

| Detailed list of the 23 ECM degradation relating genes | | | | |
| --- | --- | --- | --- | --- |
| COL11A1 | COL10A1 | TNNC1 | CTHRC1 | ABI3BP |
| PCOLCE2 | OGN | MMP1 | ADAMTS8 | SFTPC |
| GREM1 | SFTPA2 | SFTPD | WIF1 | CHRDL1 |
| CXCL2 | CXCL13 | IL6 | HHIP | CPB2 |
| MAMDC2 | LPL | CD36 |  |  |

# 1.3 Supplementary Table 3. The 27 selected HRR related genes for evaluating the association with ARHGAP44 gene

| Detailed list of the 27 HRR relating genes | | | | |
| --- | --- | --- | --- | --- |
| ATM | BARD1 | BRCA1 | BRCA2 | BRIP1 |
| CDH1 | CDK12 | CHEK1 | CHEK2 | FANCA |
| FANCL | HDAC2 | PALB2 | PPP2R2A | PTEN |
| RAD51B | RAD51C | RAD51D | RAD54L | TP53 |
| ATRX | ADRID1A | BARD1 | BLM | MRE11 |

**1.4 Supplementary Table 4. The 14 cell proliferation and 199 G2M gene checkpoints applied for evaluating the association between ARHGAP44 and cell cycle regulation**

| List of the cell proliferation relating genes | | | | | | |
| --- | --- | --- | --- | --- | --- | --- |
| Cell proliferation relating genes | MKI67 | NDC80 | PTTG1 | BIRC5 | CCND1 | CDK4 |
|  | CCNB1 | CEP55 | UBE2C | TYMS | CDKN1B | VCL |
|  | CDK6 | KRAS |  |  |  |  |
| Cell cycle G2M checkpoints | ABL1 | AC027237.1 | AC091021.1 | AMD1 | ARID4A | ATF5 |
|  | ATRX | AURKA | AURKB | BARD1 | BCL3 | BIRC5 |
|  | BRCA2 | BUB1 | BUB3 | CASP8AP2 | CBX1 | CCNA2 |
|  | CCNB2 | CCND1 | CCNF | CCNT1 | CDC20 | CDC25A |
|  | CDC25B | CDC27 | CDC45 | CDC6 | CDC7 | CDK1 |
|  | CDK4 | CDKN1B | CDKN2C | CDKN3 | CENPA | CENPE |
|  | CENPF | CHAF1A | CHEK1 | CHMP1A | CKS1B | CKS2 |
|  | CTCF | CUL1 | CUL3 | CUL4A | CUL5 | DBF4 |
|  | DDX39A | DKC1 | DMD | DR1 | DTYMK | E2F1 |
|  | E2F2 | E2F3 | E2F4 | EFNA5 | EGF | ESPL1 |
|  | EWSR1 | EXO1 | EZH2 | FANCC | FBXO5 | FOXN3 |
|  | G3BP1 | GINS2 | GSPT1 | H2AFV | H2AFX | H2AFZ |
|  | HIF1A | HIRA | HIST1H2BK | HMGA1 | HMGB3 | HMGN2 |
|  | HMMR | HNRNPD | HNRNPU | HOXC10 | HSPA8 | HUS1 |
|  | ILF3 | INCENP | JPT1 | KATNA1 | KIF11 | KIF15 |
|  | KIF20B | KIF22 | KIF2C | KIF4A | KIF5B | KMT5A |
|  | KNL1 | KPNA2 | KPNB1 | LBR | LIG3 | LMNB1 |
|  | MAD2L1 | MAP3K20 | MAPK14 | MARCKS | MCM2 | MCM3 |
|  | MCM5 | MCM6 | MEIS1 | MEIS2 | MKI67 | MNAT1 |
|  | MT2A | MTF2 | MYBL2 | MYC | NASP | NCL |
|  | NDC80 | NEK2 | NOLC1 | NOTCH2 | NSD2 | NUMA1 |
|  | NUP50 | NUP98 | NUSAP1 | ODC1 | ODF2 | ORC5 |
|  | ORC6 | PAFAH1B1 | PBK | PDS5B | PLK1 | PLK4 |
|  | PML | POLA2 | POLE | POLQ | PRIM2 | PRC1 |
|  | PRMT5 | PRPF4B | PTTG1 | PTTG3P | RACGAP1 | PURA |
|  | RAD21 | RAD23B | RAD54L | RASAL2 | RBM14 | RBL1 |
|  | RPA2 | RPS6KA5 | SAP30 | SFPQ | SLC38A1 | SLC12A2 |
|  | SLC7A1 | SLC7A5 | SMAD3 | SMARCC1 | SMC2 | SMC1A |
|  | SMC4 | SNRPD1 | SQLE | SRSF1 | SRSF2 | SRSF10 |
|  | STAG1 | STIL | STMN1 | SUV39H1 | TACC3 | SYNCRIP |
|  | TENT4A | TFDP1 | TGFB1 | TLE3 | TNPO2 | TMPO |
|  | TOP1 | TOP2A | TPX2 | TRA2B | TROAP | TRAIP |
|  | TTK | UBE2C | UBE2S | UCK2 | UPF1 | WRN |
|  | XPO1 | YTHDC1 |  |  |  |  |

**1.5 Supplementary Table 5. The 14 selected EMT related genes for evaluating the association with ARHGAP44 gene**

| List of the 14 cancers EMT relating genes | | | | |
| --- | --- | --- | --- | --- |
| VIMENTIN | CDH2 | FOXC2 | SNAI1 | SNAI2 |
| TWIST1 | FN1 | ITGB6 | MMP2 | MMP3 |
| MMP9 | GSC | CDH1 | DSP |  |

# Supplementary Figures

**
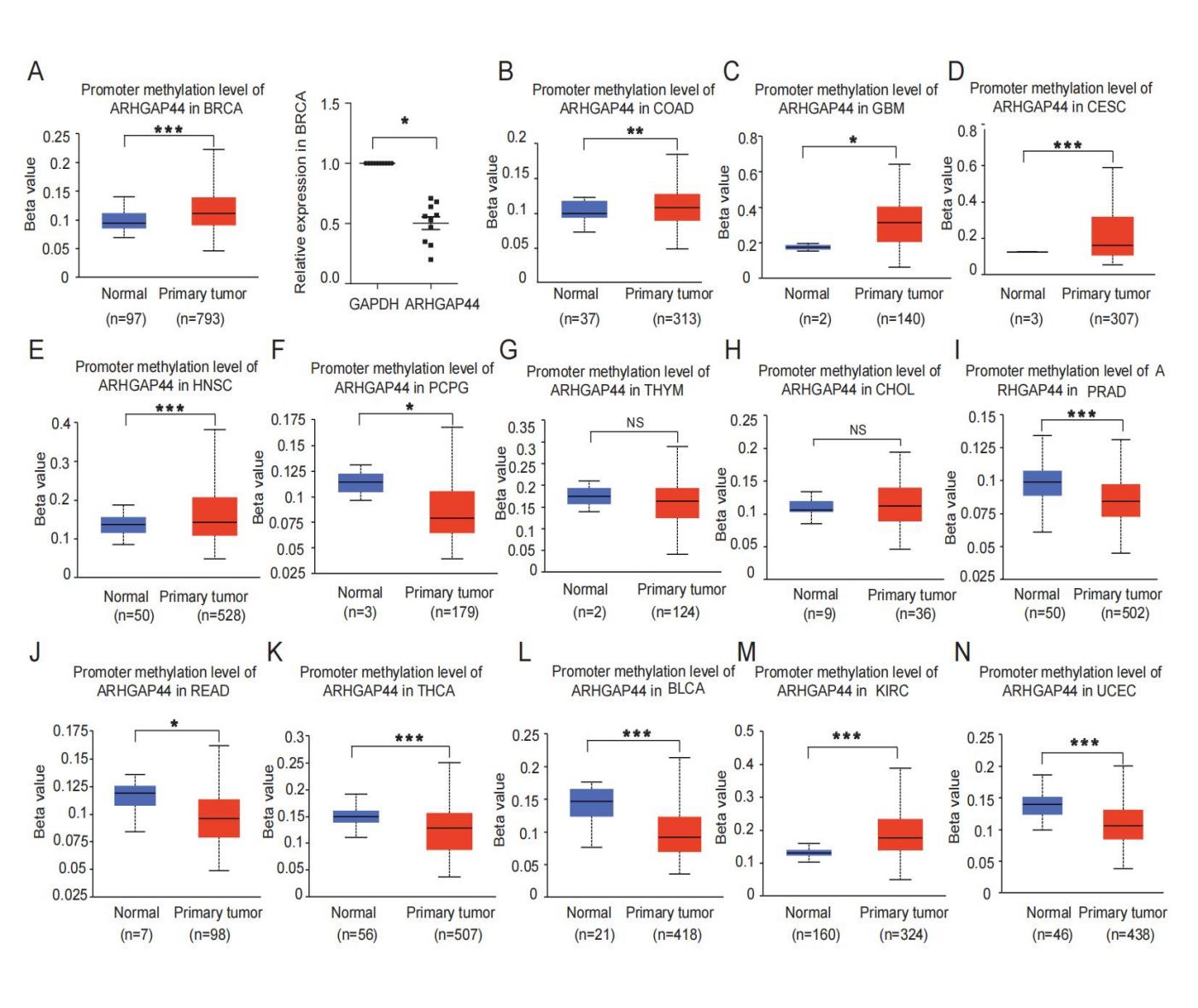
**

**Supplementary Figure 1.** The promoter methylation level of ARHGAP44 gene in (A) BRCA (right graphic indicates the QPCR detection result of ARHGAP44 gene expression in BRCA comparing to normal breast tissues), (B) KIRC, (C) COAD, (D) GBM, (E) CESC, (F) HNSC, (G) PCPG, (H) THYM, (I) CHOL, (J)PRAD, (K) READ, (L) THCA, (M) BLCA and (N) UCEC comparing to in corresponding normal samples. (*p<0.05, **p<0.01, ***p<0.001)

**
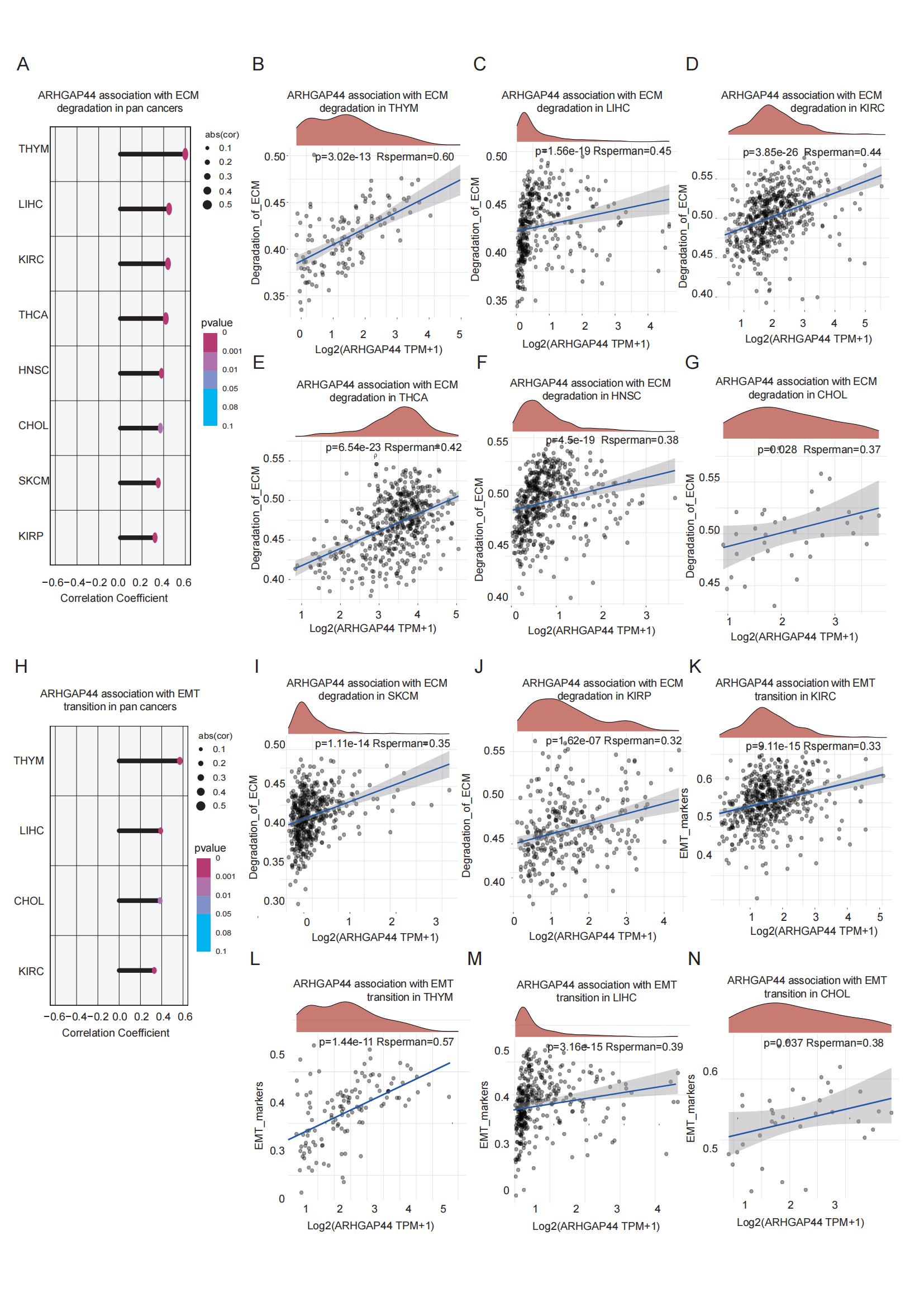
**

**Supplementary Figure 2.** ARHGAP44 association with ECM degradation related gene signature in different cancers, including in (B) THYM, (C) LIHC, (D) KIRC, (E) THCA, (F) HNSC, (G) CHOL, (I) SKCM and (J) KIRP. (H) ARHGAP44 association with EMT transition related gene signature in multiple cancers, including in (K) KIRC, (L) THYM, (M) LIHC and (N) CHOL. (R between 0.30~0.49 was considered preliminary correlated, and 0.50~0.69 was moderate correlated, meanwhile, R>0.70 was thought as strongly correlated).

**
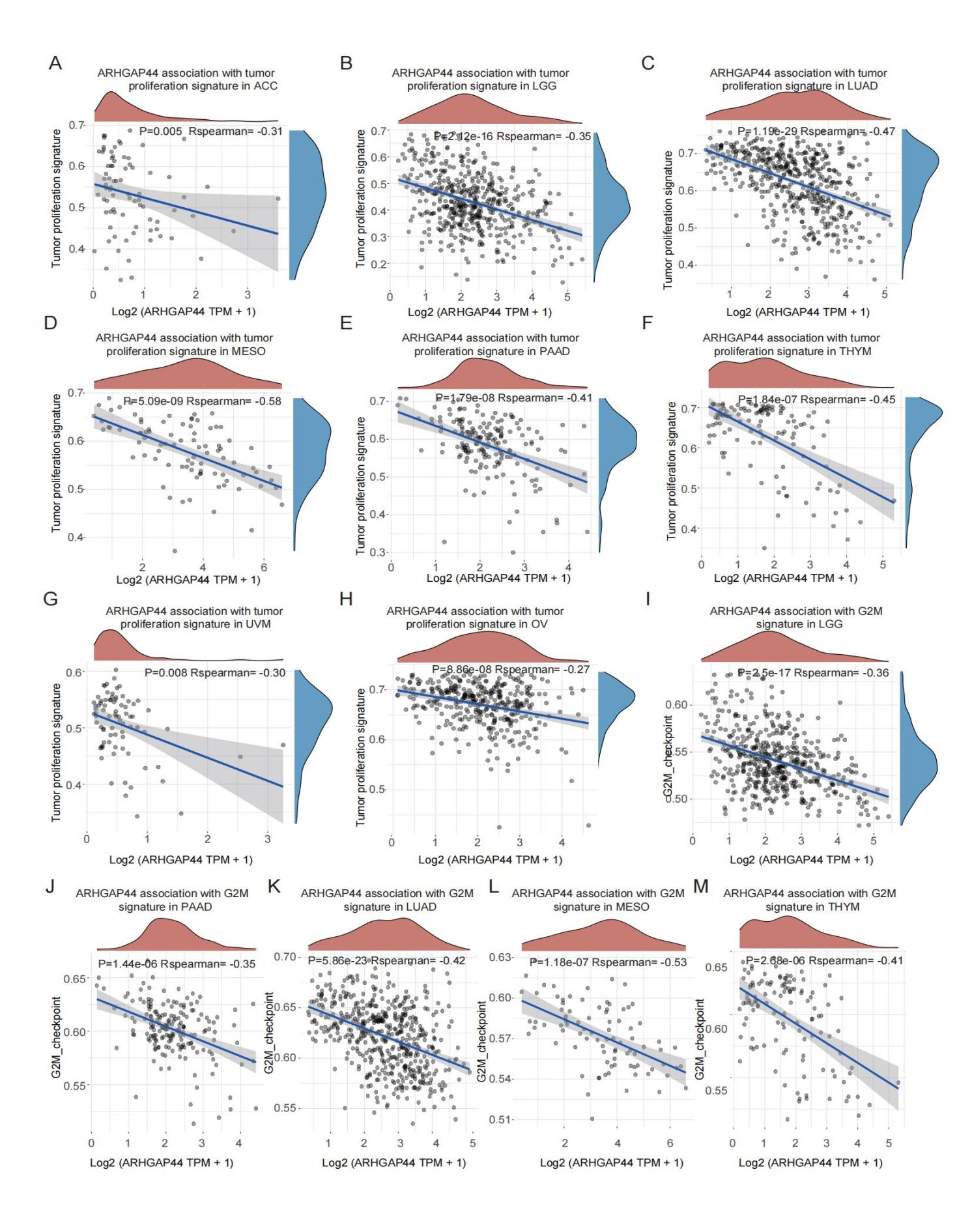
**

**Supplementary Figure 3.** ARHGAP44 association with tumor proliferation related gene signature in (A) ACC, (B) LGG, (C) LUAD, (D) MESO, (E) PAAD, (F) THYM, (G) UVM and (H) OV. ARHGAP44 association with G2M checkpoints related gene signature in (I) LGG, (J) PAAD, (K) LUAD, (L) MESO and (M) THYM. (R between 0.30~0.49 was considered preliminary correlated, and 0.50~0.69 was moderate correlated, meanwhile, R>0.70 was thought as strongly correlated).

**
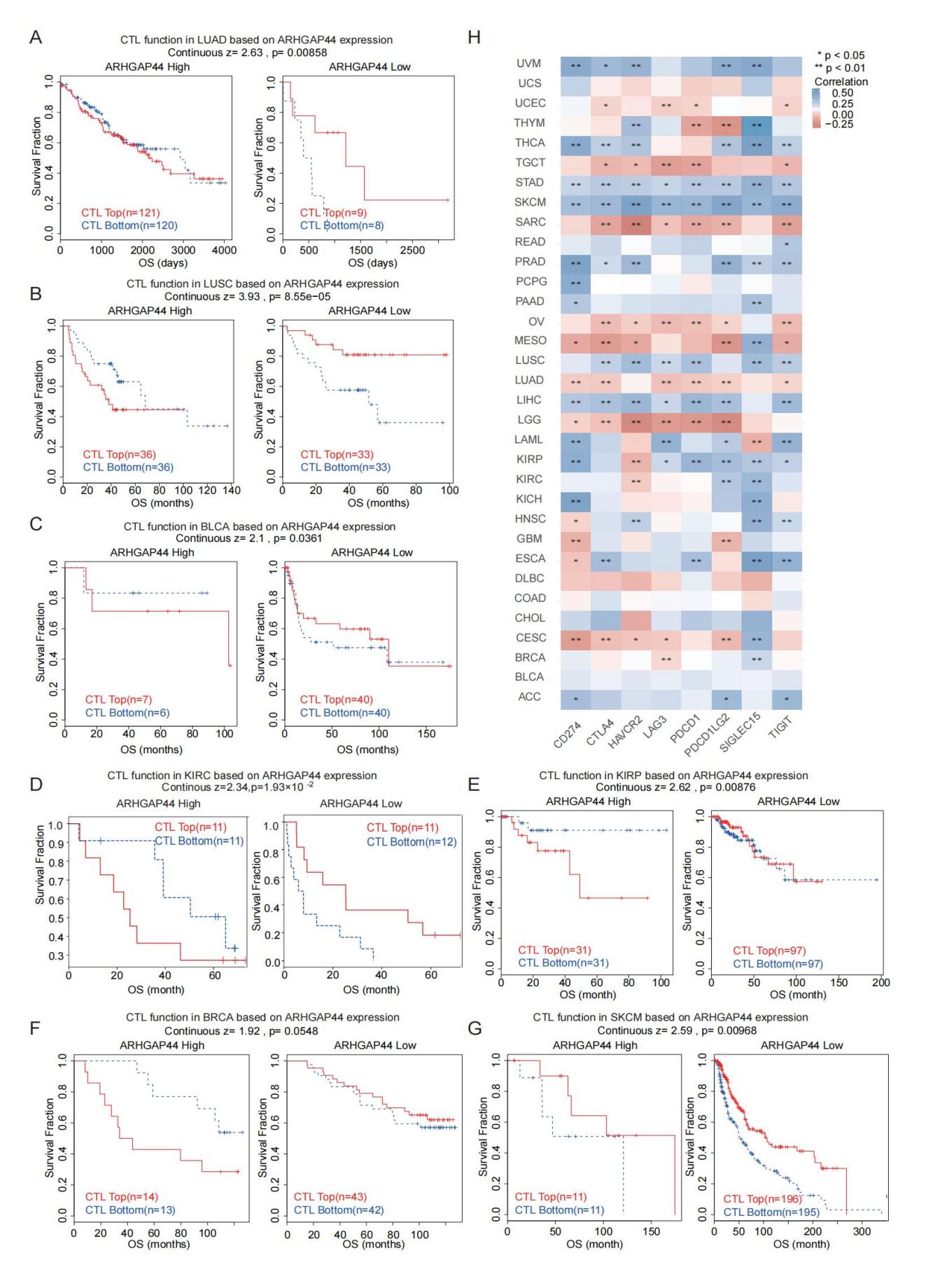
**

**Supplementary Figure 4.** Association between ARHGAP44 gene CTL function status in (A) LUAD, (B) LUSC, (C) BLCA, (D) KIRC, (E) KIRP, (F) BRCA and (G) SKCM. (H) Association between ARHGAP44 gene expression and immune checkpoints expression in pan-cancer. (p<0.05 was considered statistical significant.)
